# *Pseudomonas syringae* effector HopZ3 suppresses the bacterial AvrPto1–tomato PTO immune complex via acetylation

**DOI:** 10.1371/journal.ppat.1010017

**Published:** 2021-11-01

**Authors:** Joanna Jeleńska, Jiyoung Lee, Andrew J. Manning, Donald J. Wolfgeher, Youngjoo Ahn, George Walters-Marrah, Ivan E. Lopez, Lissette Garcia, Sheri A. McClerklin, Richard W. Michelmore, Stephen J. Kron, Jean T. Greenberg

**Affiliations:** 1 Department of Molecular Genetics and Cell Biology, The University of Chicago, Chicago, Illinois, United States of America; 2 The Genome Center & Department of Plant Sciences, University of California, Davis, California, United States of America; Texas A & M University, UNITED STATES

## Abstract

The plant pathogen *Pseudomonas syringae* secretes multiple effectors that modulate plant defenses. Some effectors trigger defenses due to specific recognition by plant immune complexes, whereas others can suppress the resulting immune responses. The HopZ3 effector of *P*. *syringae* pv. syringae B728a (*Psy*B728a) is an acetyltransferase that modifies not only components of plant immune complexes, but also the *Psy* effectors that activate these complexes. In Arabidopsis, HopZ3 acetylates the host RPM1 complex and the *Psy* effectors AvrRpm1 and AvrB3. This study focuses on the role of HopZ3 during tomato infection. In *Psy*-resistant tomato, the main immune complex includes PRF and PTO, a RIPK-family kinase that recognizes the AvrPto effector. HopZ3 acts as a virulence factor on tomato by suppressing AvrPto1_*Psy*_-triggered immunity. HopZ3 acetylates AvrPto1_*Psy*_ and the host proteins PTO, SlRIPK and SlRIN4s. Biochemical reconstruction and site-directed mutagenesis experiments suggest that acetylation acts in multiple ways to suppress immune signaling in tomato. First, acetylation disrupts the critical AvrPto1_*Psy*_-PTO interaction needed to initiate the immune response. Unmodified residues at the binding interface of both proteins and at other residues needed for binding are acetylated. Second, acetylation occurs at residues important for AvrPto1_*Psy*_ function but not for binding to PTO. Finally, acetylation reduces specific phosphorylations needed for promoting the immune-inducing activity of HopZ3’s targets such as AvrPto1_*Psy*_ and PTO. In some cases, acetylation competes with phosphorylation. HopZ3-mediated acetylation suppresses the kinase activity of SlRIPK and the phosphorylation of its SlRIN4 substrate previously implicated in PTO-signaling. Thus, HopZ3 disrupts the functions of multiple immune components and the effectors that trigger them, leading to increased susceptibility to infection. Finally, mass spectrometry used to map specific acetylated residues confirmed HopZ3’s unusual capacity to modify histidine in addition to serine, threonine and lysine residues.

## Author summary

By secreting virulence proteins (effectors) into their hosts, pathogenic bacteria hijack host cellular processes to promote bacterial colonization and disease development. For the plant pathogen *Pseudomonas syringae*, the coordinated action of effectors often mediates modifications of host defense proteins to inhibit their function. However, plants have evolved the ability to induce innate immunity upon recognition of effector-induced modifications of host proteins. How do pathogens circumvent the immune-inducing activity of certain effectors? They deploy more effectors to suppress these defenses. HopZ3, an acetyltransferase from *P*. *syringae*, is unique among plant pathogen effectors characterized so far in its ability to modify not only multiple components of the effector-triggered immune pathway, but also the triggering effector itself. Through the direct acetylation of residues involved in the interaction and activation of the bacterial effector AvrPto1_*Psy*_ and tomato kinase PTO, HopZ3 modifications disrupt their binding and block phosphorylations necessary for immune induction. Additionally, HopZ3 acetylates other possible components in the PTO signaling pathway, including activation sites in SlRIPK kinase, leading to suppression of its activity and reduced phosphorylation of SlRIN4s. Our study emphasizes the importance of HopZ3-dependent acetylation of immune complexes and bacterial effectors across plant species in the suppression of effector-induced immunity.

## Introduction

The plant pathogen *Pseudomonas syringae* uses type III-secreted proteins to promote its growth during infection of plants. These effector proteins are injected into plant cells, where they often interfere with plant defense signaling either through binding, post-translational modifications (PTMs) and/or destabilization of host factors [1,2]. A major mechanism to suppress *P*. *syringae* growth is signaling mediated by plant immune receptors that monitor specific perturbations caused by effectors. A well-studied example of such a receptor is Arabidopsis RESISTANCE TO P. SYRINGAE MACULICOLA 1 (RPM1), a member of the NUCLEOTIDE BINDING-LEUCINE RICH REPEAT (NB-LRR) protein family. Recognition and signaling occur when RPM1 senses a specific phosphorylation (mainly p-T166) of RPM1-INTERACTING PROTEIN 4 (RIN4), an intrinsically disordered hub protein [3]. Two unrelated effectors, AvrB or AvrRpm1, from different *P*. *syringae* strains can strongly trigger RPM1 signaling and are thus considered avirulence factors. These effectors cause the cytoplasmic RIN4-INDUCED PROTEIN KINASE (RIPK) and probably additional kinases to phosphorylate RIN4. RIN4 is also involved in promoting defense signaling in response to conserved microbial patterns. Immune responses are induced by phosphorylations of specific RIN4 residues that are triggered by recognition of effectors or microbial patterns [3–6].

*Pseudomonas syringae* pv. *syringae* B728a (*Psy*B728a) is a bean pathogen that can also grow to moderate levels on Arabidopsis and tomato without causing overt disease symptoms [7,8]. In Arabidopsis, *Psy*B728a with a deletion of the type III secreted effector HopZ3 (*Psy*ΔHopZ3) causes the activation of RPM1 signaling. This occurs via two interacting effectors with homology to AvrB and AvrRpm1: AvrB3_*Psy*_ and AvrRpm1_*Psy*_. In the context of *Psy*ΔHopZ3 infection, both effectors are needed to activate signaling [9]. HopZ3 belongs to the YopJ acetyltransferase family that comprises several effectors from animal and plant pathogens. The acetyltransferase activity of HopZ3 is necessary for suppression of RPM1 activation in Arabidopsis and several components of the RPM1 immune-effector complex are substrates of HopZ3 [9]. HopZ3 acetylates the activation loop and active site residues of RIPK, which inhibits its ability to phosphorylate RIN4. Additionally, acetylation of RIN4 prevents its phosphorylation by RIPK. HopZ3 also acetylates residues in AvrB3 that are predicted to disrupt hydrogen bonds at the key interaction sites with RIN4. Thus, HopZ3 suppresses plant immunity through modification of both Arabidopsis and bacterial proteins that act in the same complex.

Interestingly, in a large screen for interactions between effectors and plant immune signaling proteins ([9], https://charge.ucdavis.edu/charge_db/interaction/Y2H/Y2H_interaction.php), we found that HopZ3 interacted with the resistance-inducing effector AvrPto1_*Psy*_ and its tomato targets, PTO-like proteins. Moreover, HopZ3 suppressed AvrPto1_*Psy*_-induced cell death in *Nicotiana benthamiana* [8]. That suggested that HopZ3 may affect tomato immunity. The interaction between *Psy*B728a and tomato has not been well characterized; however, resistance to *P*. *syringae* pv. *tomato* has been studied in great detail. Resistant tomato lacks RPM1 but contains PSEUDOMONAS RESISTANCE AND FENTHION SENSITIVITY (PRF), an NB-LRR protein that forms complexes with the kinases PSEUDOMONAS SYRINGAE PV TOMATO RESISTANCE (PTO) and FENTHION SENSITIVITY (FEN) and recognizes effectors AvrPto and AvrPtoB from *P*. *syringae* pv. *tomato* and other pathovars [10]. PTO, FEN and related cytoplasmic protein kinases in the same family as RIPK show natural variation that affects their functional specificity in promoting immunity in different tomato accessions [11]. PTO and FEN interact differently with AvrPto and AvrPtoB. Both effectors can bind to PTO and elicit PRF-dependent immune signaling [12–15]. In contrast, FEN can bind and be activated by AvrPto if the key residue N202 (that corresponds to T204 in PTO) is substituted with threonine [16]. Truncated versions of AvrPtoB (e.g., AvrPtoB_1-387_) bind to FEN and stimulate immunity; however, due to the C-terminal E3 ubiquitin ligase domain, full-length AvrPtoB causes proteasome-dependent FEN degradation and does not trigger FEN/PRF immunity [14]. Structure-based biochemical analysis has indicated that AvrPto-PTO binding is a key step that leads to activation of PRF signaling [17]. The kinase activity of PTO is important for disease resistance triggered by AvrPto [18–22]. PTO acts as a dimer or higher order complex together with PRF [17,22,23]. Although AvrPto can inhibit PTO and other kinases [17], transphosphorylation between unbound PTO molecules and those bound to AvrPto is thought to be needed for downstream signaling [17,22,23].

Another potential player in PTO/PRF-conferred immunity is SlRIN4-1, one of three RIN4-related proteins in tomato. Infection with *P*. *syringae* pv. *tomato* strain T1 engineered to express AvrPto causes reduction of SlRIN4 protein levels. Downregulation of SlRIN4-1 using RNAi decreases the growth of strain T1 carrying AvrPto but not the growth of strain T1 alone [24]. Thus, downregulation of SlRIN4-1 seems to specifically enhance PTO-dependent resistance. Moreover, *N*. *benthamina* homologue of RIN4 was found in a search for proteins proximal to AvrPto, suggesting their interaction [25].

*Psy*B728a has AvrPto and AvrPtoB homologues (AvrPto1_*Psy*_ and AvrPtoB_*Psy*_/HopAB1, hereafter called AvrPtoB_*Psy*_) that induce resistance in tomato. Transfer of a plasmid carrying AvrPto1_*Psy*_ to a *P*. *syringae* pv. *syringae* strain that lacks AvrPto and AvrPtoB (*Psy*61) confers PTO-dependent recognition, whereas plasmid-borne AvrPtoB_*Psy*_ confers some PTO-independent recognition that involves other members of PTO family [26]. AvrPto1_*Psy*_ is 88% identical at the amino acid level with AvrPto_*Pto*_ while AvrPtoB alleles share 52% identity. Both AvrPto1_*Psy*_ and AvrPtoB_*Psy*_ can interact with PTO in a yeast two-hybrid assay [26]. Consistent with these findings, PRF is a major factor that restricts the growth of *Psy*B728a on tomato [10,26].

We previously found that deletion of HopZ3 decreased the growth of *Psy* on tomato with functional PTO [7], raising the possibility that HopZ3 normally suppresses effector-triggered immunity in tomato. In this study, we investigated this hypothesis. Through genetics and biochemical reconstruction, our data point to a mechanism that involves immune suppression *via* acetylation of AvrPto1_*Psy*_, PTO and other immunity factors.

## Results

### HopZ3 suppresses PTO/PRF defenses triggered by AvrPto1_*Psy*_

**Fig 1:**
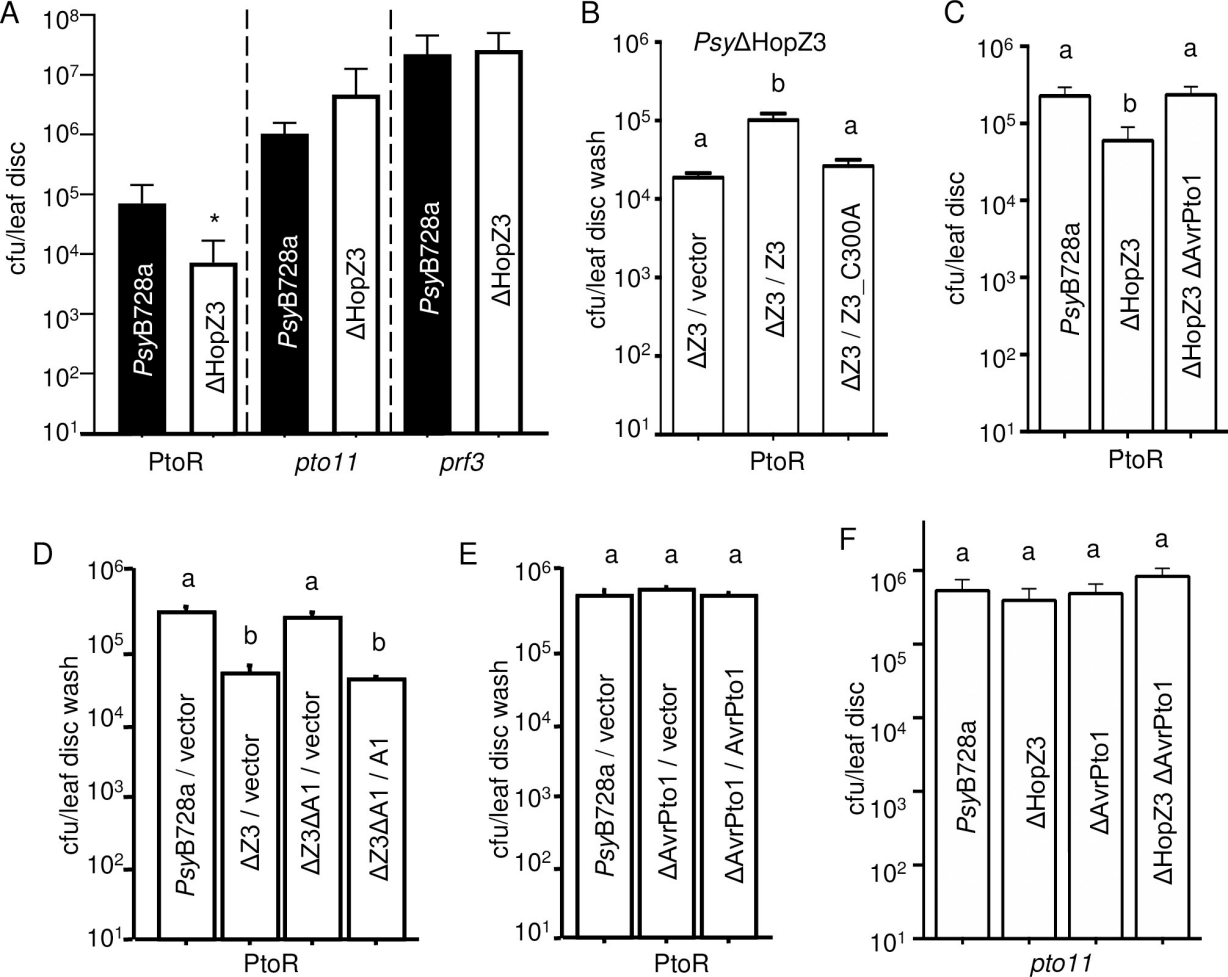
HopZ3 promotes the growth of *Psy*B728a on PTO-containing tomato plants (PtoR) and suppresses defenses triggered by AvrPto1_*Psy*_. Plants were spray inoculated with *Ps*yB728a-derived strains at an OD_600_ = 0.01 and total (epiphytic + endophytic) or epiphytic bacterial populations were quantified in 8 leaf discs or leaf disc washes, respectively. (A) Total bacterial populations of *Ps*yB728a and *Psy*ΔHopZ3 were different in PtoR but were not statistically different in *pto11* and *prf-3* plants after 4 days (n = 8, t-test *P<0.05). (B) HopZ3 (Z3), but not the catalytic mutant (Z3_C300A) complements the low growth phenotype of *Psy*ΔHopZ3 in PtoR tomato. (C-D) Deletion of AvrPto1_*Psy*_ (ΔA1) from *Psy*ΔHopZ3 (ΔZ3) restores total (C) and epiphytic (D) bacterial growth to WT (*Psy*B728a or *Psy*B728a/vector) levels in PtoR tomato. (E) Deletion of AvrPto1_*Psy*_ from WT *Psy*B728a does not affect bacterial growth in PtoR tomato. (F) AvrPto1_*Psy*_ does not confer resistance in *pto11* plants, regardless of the presence of HopZ3. For (B,D,E) epiphytic bacteria were collected by leaf disc washes five (B) or four (D-E) days after inoculation. Different letters indicate significant differences in growth as assessed by ANOVA with Tukey’s test (P<0.0002) or Fisher’s test P<0.0001, n = 8). For C and F, total bacteria were quantified 3 days after spray inoculation. Different letters indicate significant differences in growth (n = 8, ANOVA with Tukey’s test, P<0.05). All experiments were repeated at least twice with similar results. Bars indicate standard errors.

*Psy*B728a has a strong epiphytic growth phase modulated by effectors [7]. *P*. *syringae* effectors, including AvrPto_*Pto*_, are predominantly expressed by bacteria on a leaf surface and delivered to epidermal cells during infection, where they can induce and suppress defenses [7,27]. Deletion of HopZ3 reduced epiphytic growth of *Psy*B728a in a resistant tomato PtoR (76R), which has a functional PTO [7]. In a transient expression assay in *N*. *benthamiana*, HopZ3 suppressed AvrPto1_*Psy*_-induced cell death, a proxy for immune activation [7,8]. Therefore, it seemed plausible that the effect of HopZ3 on the growth of *Psy*B728a in tomato is dependent on PTO and PRF proteins needed for recognition and resistance triggered by AvrPto1_*Psy*_. Bacterial growth of *Psy*B728a and *Psy*ΔHopZ3 was indistinguishable in *pto11* and *prf3* plants lacking functional PTO and PRF, respectively, indicating that the PTO/PRF pathway is needed for the effect of HopZ3 (Fig 1A). As expected, deletion of HopZ3 similarly restricted total (epiphytic + endophytic, Fig 1A and 1C) and epiphytic (Fig 1B and 1D) populations of *Psy*B728a in PtoR tomato and we tested these populations interchangeably in further experiments. The growth defect of *Psy*ΔHopZ3 was restored only when a plasmid carrying wild-type HopZ3 but not a catalytically inactive version (HopZ3_C300A) was introduced (Fig 1B). HopZ3 and HopZ3_C300A proteins in these strains are produced at the same level in *Psy*ΔHopZ3 [7]. These results suggest that enzymatically active HopZ3 suppresses PTO-mediated plant immunity in tomato.

A possible explanation for why PTO is needed to observe HopZ3’s effect on promoting *Psy*B728a growth is that HopZ3 suppresses AvrPto1_*Psy*_ recognition. If this is true, the effect of deleting HopZ3 should be reversed when AvrPto1_*Psy*_ is also deleted. To test this hypothesis, we assessed the growth of a double mutant of *Psy*B728a that lacks both HopZ3 and AvrPto1_*Psy*_ in PtoR tomato. Both total (Fig 1C) and epiphytic (Fig 1D) populations of *Psy*ΔHopZ3ΔAvrPto1_*Psy*_ were increased relative to *Psy*ΔHopZ3 to levels similar to WT *Psy*B728a. The effect of deleting AvrPto1_*Psy*_ was complemented when the double mutant was transformed with a plasmid carrying AvrPto1_*Psy*_ (Fig 1D). Deletion of AvrPto1_*Psy*_ in *Psy*B728a with intact HopZ3 had no effect on the growth of *Psy*B728a in PtoR tomato (Fig 1E), as previously reported [28]. AvrPto1_*Psy*_ did not confer resistance in *pto11* plants due to lack of functional PTO, regardless of the presence of HopZ3 (Fig 1F). Altogether, our genetic analysis indicates that HopZ3 suppresses AvrPto1_*Psy*_-triggered immunity during *Psy*B728a infections.

### HopZ3 interacts with SlRIN4s, tomato kinases PTO, FEN, SlRIPK and effectors that target PTO

**Fig 2:**
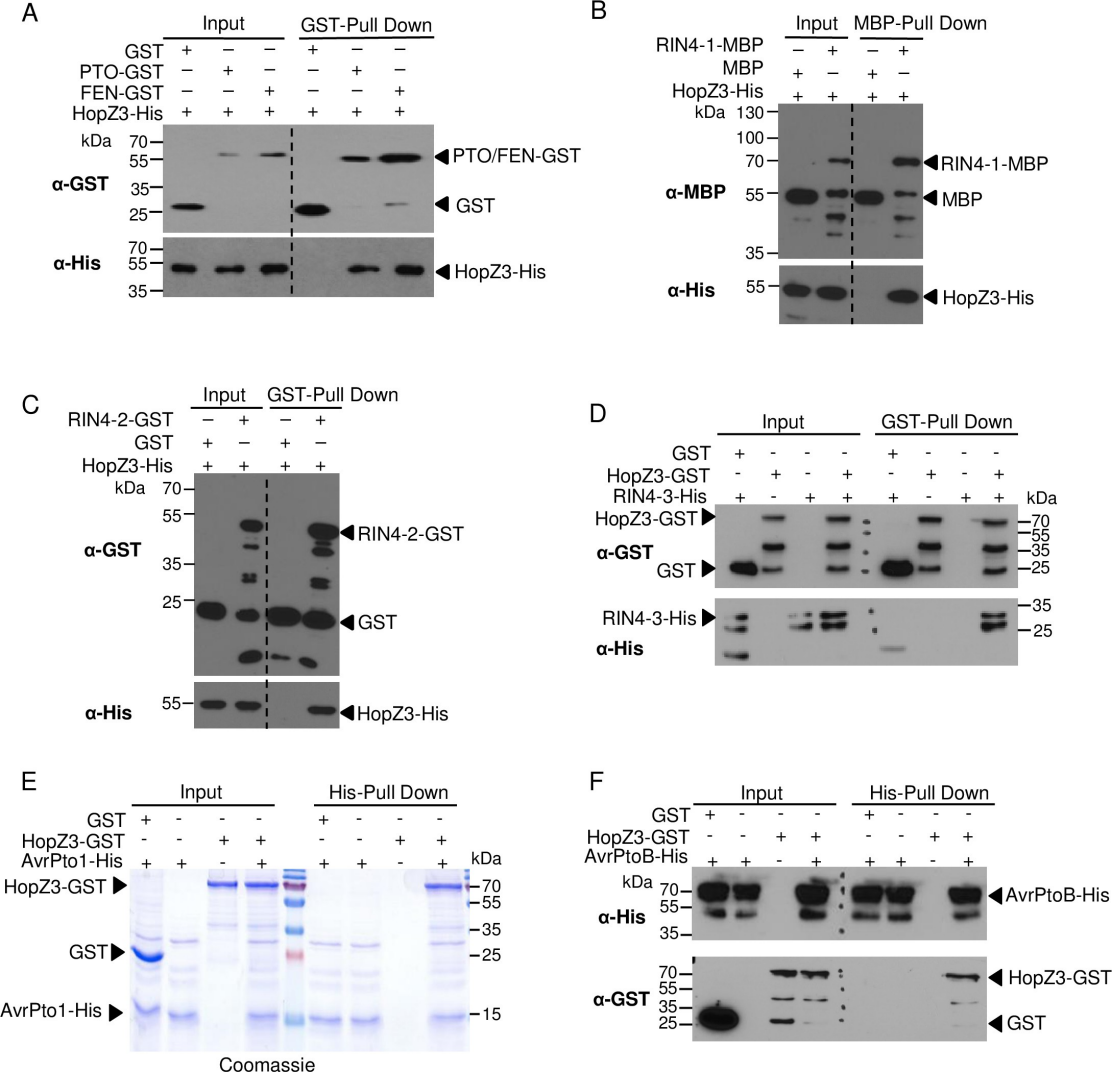
HopZ3 interacts with members of the PTO defense pathway and PTO activating effectors. Pull-down experiments using recombinant tagged proteins were performed to assess the interaction between HopZ3 and proteins in the PTO immune pathway. GST, MBP or beads alone were used as negative controls. Proteins were detected by immunoblotting or Coomassie staining. (A) Immobilized GST-tagged FEN and PTO were incubated with purified HopZ3-His, washed and resolved by SDS-PAGE. Immunoblotting showed interactions between these proteins and HopZ3. (B-D) HopZ3 interacts with all three tomato RIN4s. Immobilized MBP-tagged SlRIN4-1 (B) or GST-tagged SlRIN4-2 (C) pulled down HopZ3-His and immobilized HopZ3-GST pulled down SlRIN4-3-His (D). (E) GST-tagged HopZ3 was pulled down using immobilized AvrPto1_*Psy*_-His, showing their interaction. (F) HopZ3-GST was pulled down by immobilized AvrPtoB_*Psy*_-His. Dashed lines in (A-C) separate input from pull down in the same blot and in (D, F) they mark protein weight standards.

To investigate the molecular mechanisms of HopZ3 suppression of tomato immunity, we performed a screen for HopZ3 and AvrPto1_*Psy*_ interacting proteins using a semi-automated yeast two-hybrid analysis ([9], https://charge.ucdavis.edu/charge_db/interaction/Y2H/Y2H_interaction.php). Initial yeast experiments indicated interactions of HopZ3 with SlRIN4-1, SlRIN4-2, PTO homologous protein2 (PTH2), PTO homologous protein4 (PTH4), FEN, AvrPto1_*Psy*_ and AvrPtoB_*Psy*_. We followed up on a subset of these proteins and also tested additional candidate proteins (S1 Fig and Table 1). Although HopZ3 and PTO did not show an interaction in the yeast two-hybrid assays ([7]; S1 Fig), they interacted in an *in vitro* pull-down assay and *in planta* bimolecular fluorescence complementation (BIFC) analysis (Table 1 and Figs 2A and S2). In addition, HopZ3 interacted with FEN, tomato RIN4 homologues (SlRIN4-1, -2 and -3), the bacterial effectors AvrPto1_*Psy*_ and AvrPtoB_*Psy*_ in *in vitro* pull-downs and *in planta* and with SlRIPK in yeast and *in planta* (Figs 2 and S1 and S2 and Table 1).

**Table 1:** HopZ3 interacts with members of a tomato immune complex.

	Yeast two-hybrid		*In vitro* pull-down		BIFC in *N*. *benthamiana*	
	HopZ3	AvrPto1_*Psy*_	HopZ3	AvrPto1_*Psy*_	HopZ3	AvrPto1_*Psy*_
SlPTO	-	+	+	+	+	weak
SlFEN	weak	+	+	-	weak	-
SlRIN4-1	+	weak	+	-/weak	+	+
SlRIN4-2	+	weak	+	weak	+	+
SlRIN4-3	nd	nd	+	-	+	+
SlRIPK	weak	+	nd	nd	+	+
AvrPto1_*Psy*_	+	+	+	nd	+	weak
AvrPtoB_*Psy*_	+	+	+	+	+	-/weak

Interacting partners of HopZ3 and AvrPto1_*Psy*_ in yeast two-hybrid analysis, *in vitro* pull-down and *in planta* BIFC are shown. + indicates interaction; weak indicates weak signal; -, no interaction (no signal); nd, not determined. When interactions were tested in two directions, the stronger score is reported in the table. See also Figs 2 and S1, S2 and S3 for details of interactions in different tests and additional combinations.

HopZ3 and AvrPto1_*Psy*_ displayed similar protein–protein interaction profiles. AvrPto1_*Psy*_ directly interacted with the same tomato kinases and SlRIN4s as HopZ3 in at least one of the assays (Table 1 and S1–S3 Figs), which suggests these proteins are common targets for both effectors. As expected, recombinant AvrPto1_*Psy*_ could directly bind to PTO *in vitro* (Table 1 and S3A Fig), similarly to what was shown for AvrPto_*Pto*_ [17]. We also detected a weak signal using BIFC in *N*. *benthamiana*, suggesting *in planta* complex formation of AvrPto1_*Psy*_ and PTO (Table 1 and S2 Fig). However, AvrPto1_*Psy*_ did not show interaction with FEN *in vitro* or *in planta* (Table 1 and S2 and S3B Figs). In addition to HopZ3, AvrPto1_*Psy*_ also interacted with AvrPtoB_*Psy*_ in yeast two-hybrid and *in vitro* pull-down assays (Table 1 and S1 and S3F Figs). Many of HopZ3 interacting proteins interacted with each other (S1 and S2 Figs). These data show that HopZ3 directly targets the AvrPto-PTO defense pathway in tomato.

### HopZ3 acetylates a subset of interacting proteins

**Fig 3:**
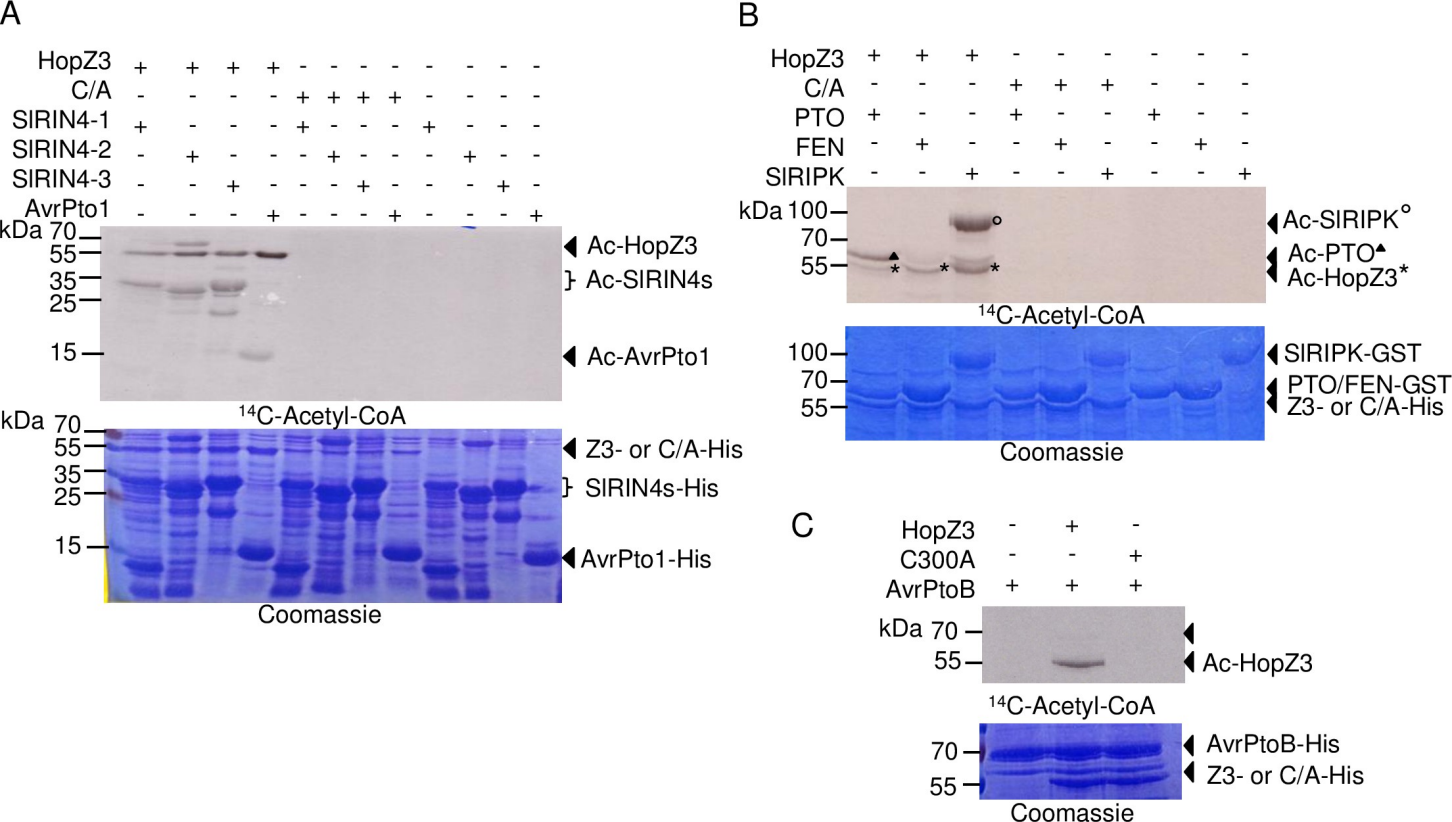
HopZ3 acetylates SlRIN4-1,-2, -3, AvrPto1_*Psy*_, PTO, SlRIPK but not FEN or AvrPtoB_*Psy*_. Purified recombinant His-tagged SlRIN4-1, -2, -3, AvrPto1_*Psy*_, AvrPtoB_*Psy*_ and GST-tagged PTO, FEN and SlRIPK proteins were incubated with His-tagged HopZ3 or HopZ3_C300A mutant (C/A) in the presence of IP6 and ^14^C-acetyl-CoA for 2 h at 30°C. Samples were separated by SDS-PAGE and subjected to autoradiography for 14 days. (A) SlRIN4-1, -2, -3 and AvrPto1_*Psy*_ were acetylated by HopZ3. (B) PTO and SlRIPK were acetylated by HopZ3; however, FEN acetylation was not detected. (C) AvrPtoB_*Psy*_ was not significantly acetylated by HopZ3.

Since HopZ3 has acetyltransferase activity [9], we tested whether several interacting proteins were its substrates *in vitro*, in reactions with ^14^C-acetyl-CoA and the cofactor inositol hexakis-phospate (IP6). Recombinant HopZ3, but not the catalytically inactive variant HopZ3_C300A, acetylated AvrPto1_*Psy*_ and its target PTO, SlRIPK, SlRIN4-1, SlRIN4-2 and SlRIN4-3 (Fig 3A and 3B). There was no detectable acetylation of FEN by HopZ3 (Fig 3B). Although AvrPtoB_*Psy*_ was capable of binding to HopZ3, it was not a good substrate for acetylation (Fig 3C). Despite diversity of substrates, HopZ3 activity is specific, as the enzyme does not acetylate interacting proteins MPK4 [9], FEN and AvrPtoB_*Psy*_ or non-interacting HopI_*Psy*_ [9].

### HopZ3 acetylates AvrPto1_*Psy*_ residues essential for interaction with PTO and decreases phosphorylation of residues involved in defense activation

**Fig 4:**
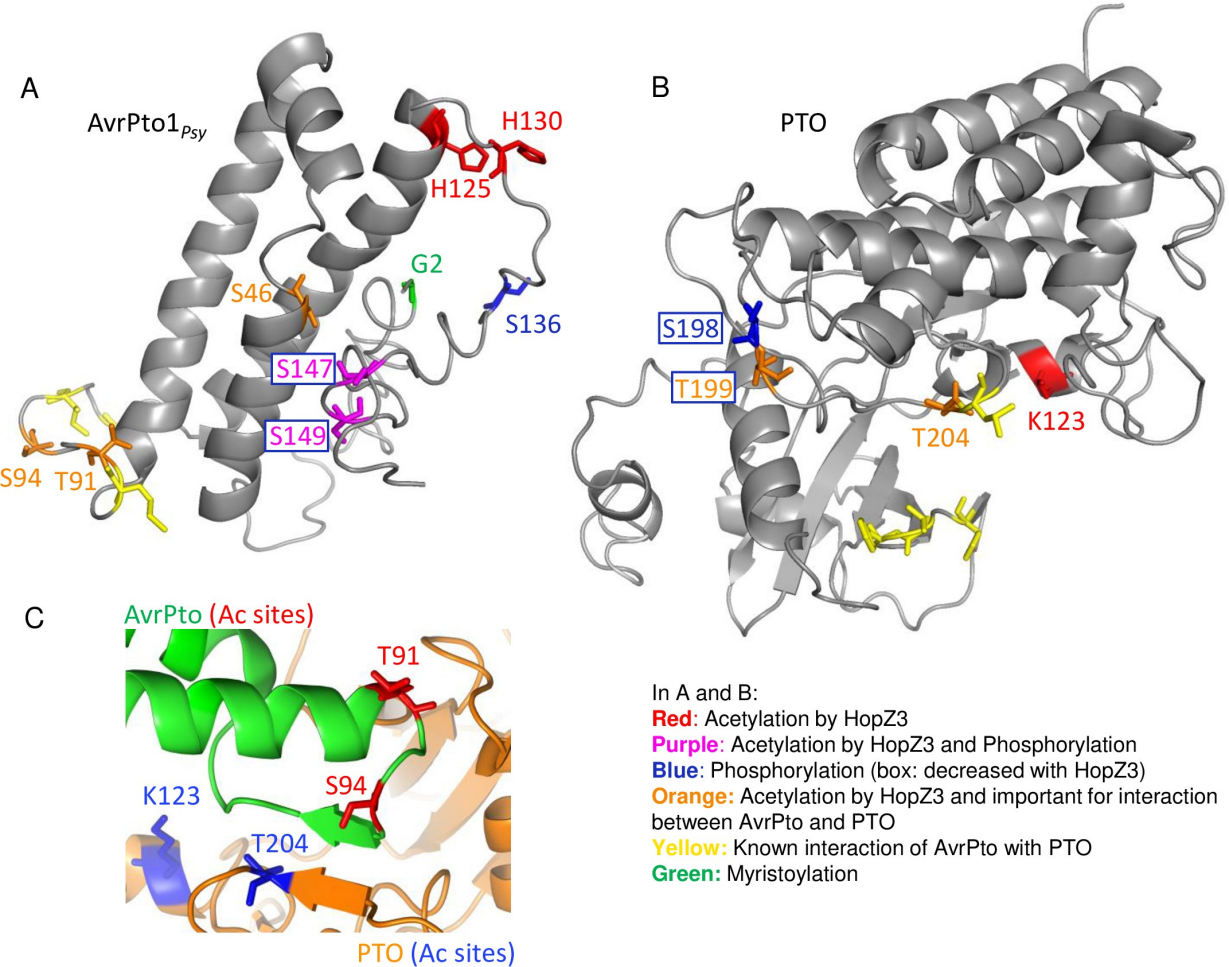
HopZ3 acetylates multiple sites in AvrPto1_*Psy*_ and PTO important for their interaction and signaling. AvrPto1_*Psy*_ and PTO co-expressed with HopZ3 or HopZ3_C300A in *N*. *benthamiana* were analyzed using mass spectrometry for post translational modifications. (A–B) Models of the AvrPto1_*Psy*_ and PTO showing the modifications identified in the *in planta* experiment that are important for immune signaling. Models were developed using the iTASSER modeling server and algorithm. Major acetylation sites dependent on HopZ3 are shown in red, important phosphorylation sites in blue, sites either acetylated or phosphorylated in purple, known sites of interaction between AvrPto_*Pto*_ and PTO in yellow, acetylated interaction sites in orange and G2 myristoylation site in green. See also S1 and S2 Tables and S4 and S5 Figs. HopZ3 acetylates sites essential for interaction (orange) and decreases phosphorylation of residue(s) involved in signaling (blue box). (C) Model of HopZ3 acetylation sites in the crystal structure of PTO:AvrPto_*Pto*_ contact site [17]. AvrPto is shown in green with residues acetylated by HopZ3 in red, and PTO is shown in orange with sites acetylated by HopZ3 in blue. Modifications on either protein are in the known interaction area of the two proteins.

To gain further insight into molecular mechanisms of immune suppression by HopZ3, we analyzed post-translational modifications of AvrPto1_*Psy*_ produced in *E*. *coli* and *N*. *bentha-miana* by LC-MS/MS. By comparing acetylation sites found in *E*. *coli*-produced AvrPto1_*Psy*_ after *in vitro* acetylation reactions with ^13^C-acetyl-CoA, IP6 and HopZ3 or HopZ3_C300A, we found that H125 and H130 were specifically acetylated by HopZ3 (S1 Table). These histidine residues were also specifically acetylated *in planta*, when AvrPto1_*Psy*_ and HopZ3 were co-expressed in *N*. *benthamiana*. Several other AvrPto1_*Psy*_ residues were acetylated *in vitro* and *in planta* to higher levels in the presence of HopZ3 compared to HopZ3_C300A (S1 Table and Figs 4 and S4). T91 and S94 in the AvrPto1_*Psy*_ GINP Ω loop that is essential for interaction with PTO [15,17,29,30] were consistently found to be the most highly acetylated in several experiments (S1 Table). S46, which is also important for interaction with PTO [15,29,30] and the virulence function of AvrPto_*Pto*_ [31], was also acetylated by HopZ3. This residue is not in the binding interface, but likely stabilizes the protein fold [30].

Many residues in AvrPto1_*Psy*_ produced in *E*. *coli* or in *N*. *benthamiana* were phosphorylated (S1 Table and Figs 4 and S4). Interestingly, S136 was very highly phosphorylated *in planta* (regardless of the presence of HopZ3), but it was not phosphorylated in the recombinant protein. This plant modification of AvrPto has not been reported previously; its functional significance is unknown and was not further explored. Since HopZ3 also targets serines and threonines, the same residues may also be phosphorylated. S147 and S149 of AvrPto1_*Psy*_ were phosphorylated *in vitro* and *in planta*, and HopZ3 acetylated a fraction of these residues as well. Importantly, in *N*. *benthamiana* expressing HopZ3, phosphorylation of S147 and/or S149 was significantly reduced (S1 Table). These residues were previously shown to be phosphorylated and contribute to the avirulence activity of AvrPto_*Pto*_ during interactions with resistant tomato [32] and *Nicotiana* sp. [33], as well as to virulence during susceptible tomato infection [32]. In our LC-MS/MS analysis, we also directly detected myristoylation of G2, a modification that enables membrane localization of AvrPto [32] (S1 Table and Figs 4 and S4).

Acetylation of residues in the AvrPto1_*Psy*_ Ω loop that interacts with PTO and decreased phosphorylation of residue(s) involved in signaling likely contribute to the mechanism by which HopZ3 reduces the immune response to AvrPto1_*Psy*_.

### Residues acetylated by HopZ3 are important for AvrPto1_*Psy*_ avirulence during tomato infection

**Fig 5:**
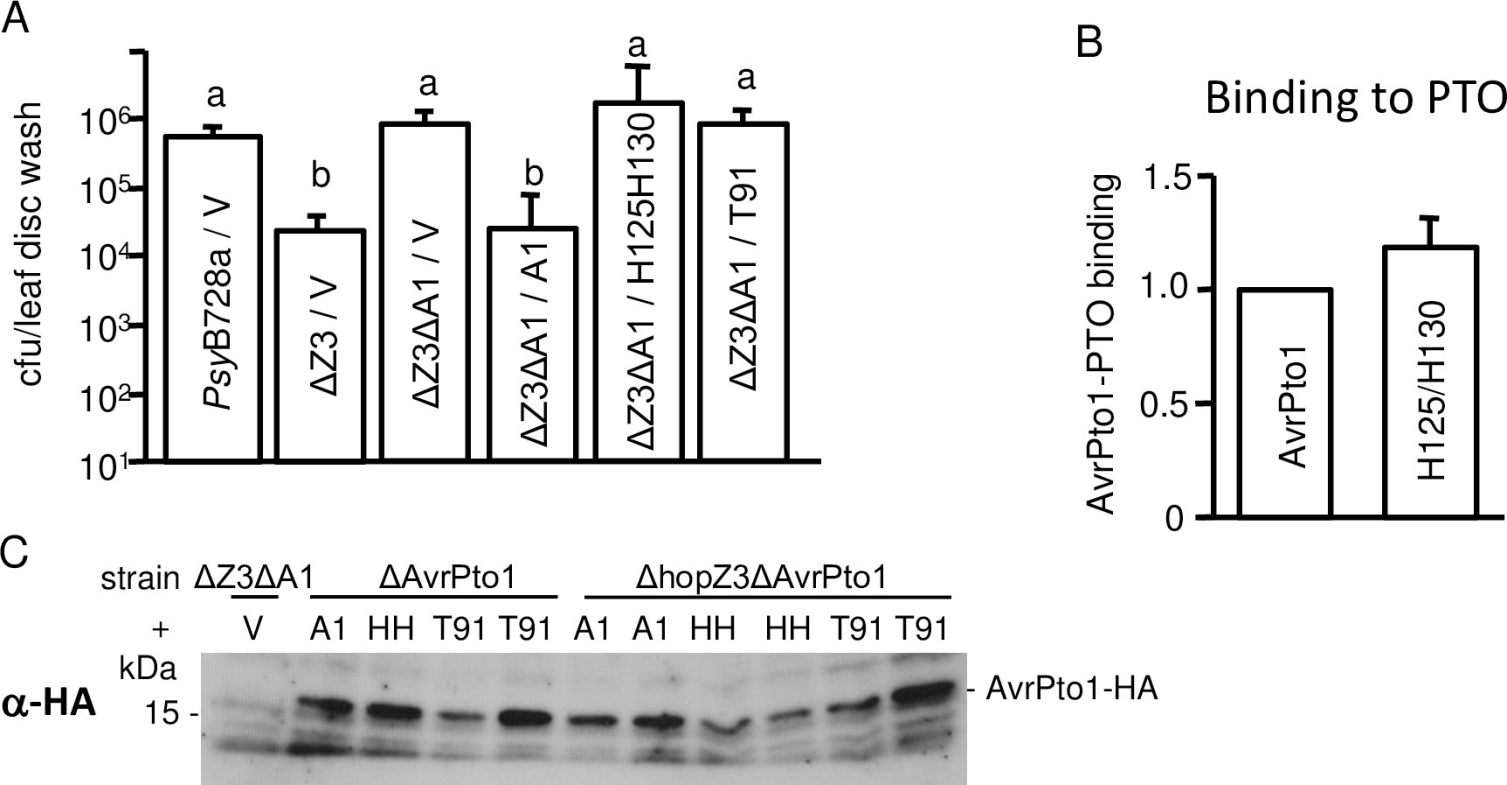
Effect of mutations of AvrPto1_*Psy*_ acetylation sites on *Psy*B728a growth in tomato. (A) AvrPto1_*Psy*__T91A and H125A/H130A mutants did not reduce *Psy*B728a growth in PTO-containing tomato in the absence of HopZ3. Plants were spray-inoculated with indicated strains at an OD_600_ = 0.01. Epiphytic bacterial populations were quantified in leaf disc washes from eight different plants per strain four days after inoculation. Different letters indicate significant differences in growth as assessed by ANOVA with Tukey’s test (P<0.05). Similar results were found in at least two other experiments (AvrPto1_*Psy*__H125A/H130A did not reduce the growth of *Psy*B728a ΔHopZ3 in three out of five experiments). Bars indicate standard errors. V, vector control; A1, AvrPto1_*Psy*_; Z3, HopZ3; T91, AvrPto1_*Psy*__T91A; H125/H130 and HH, AvrPto1_*Psy*__H125A/H130A. (B) H125A/H130A mutation of AvrPto1_*Psy*_ did not affect its binding to PTO. AvrPto1_*Psy*_-GST, AvrPto1_*Psy*__H125A/H130A-GST and PTO-MBP were expressed in *E*. *coli*. Purified soluble AvrPto1_*Psy*_ or H125A/H130A mutant was pulled down with immobilized PTO-MBP or, alternatively, soluble PTO was pulled down with immobilized AvrPto1_*Psy*_-GST or AvrPto1_*Psy*__H125A/H130A-GST. Band intensities were quantified from seven experiments. (T-test, P = 0.1). (C) AvrPto1 mutant variants were expressed to similar levels as AvrPto1_*Psy*_ in ΔAvrPto1 and ΔhopZ3ΔAvrPto1 *Psy*B728a grown in type III secretion-inducing conditions.

Many residues acetylated by HopZ3 are important for the ability of AvrPto1_*Psy*_ to trigger a defense response in resistant tomato. For example, S94 and S147/S149 in AvrPto_*Pto*_ were shown to contribute to triggering PTO-mediated disease resistance and were extensively studied, as discussed above. Although T91 in the GINP Ω loop was not found to affect interaction with PTO in any mutagenesis studies, a T91A variant that we constructed lost the ability to suppress the growth of *Psy*B728a ΔHopZ3 in PtoR tomato (Fig 5A) and was defective in the induction of cell death in *N*. *benthamiana* (S6 Fig). H125/H130 residues are on the opposite side of AvrPto1_*Psy*_ molecule from the Ω loop (Fig 4) and their substitutions did not disrupt *in vitro* binding to PTO (Fig 5B) or cell death induction in *N*. *benthamiana* (S6 Fig). Nevertheless, H125A/H130A substitutions reduced the ability of AvrPto1_*Psy*_ to suppress bacterial growth in resistant tomato (Fig 5A). Importantly, AvrPto1_*Psy*_ variants were expressed in *Psy*B728a to similar levels as wild-type AvrPto1_*Psy*_ (Fig 5C). Therefore, the residues acetylated by HopZ3 are important for the ability of AvrPto1_*Psy*_ to trigger a defense response in resistant tomato.

### HopZ3 acetylates key sites in the activation loop and other residues important for the immune function of PTO and reduces their phosphorylation

We used an LC-MS/MS analysis of PTO to gain insight into what specific effect acetylation might have. By comparing acetylation sites found in the presence of HopZ3 and HopZ3_C300A after *in vitro* acetylation reactions with ^13^C-acetyl-CoA, we identified T204 in the P+1 activation loop/region of PTO as a specific HopZ3-mediated acetylation site (S2 Table and S5 Fig). T204 is a cognate of T257 in Arabidopsis RIPK, another member of this kinase family that we found to be acetylated by HopZ3 [9].

T204 and T199 were the major acetylation sites *in planta* in PTO immunoprecipitated from *N*. *benthamiana* that also expressed functional HopZ3 (S2 Table and Figs 4 and S5). Both of these residues in the P+1 loop are important for interaction with AvrPto [16,17,20,22]. In addition, the structurally proximal residue K123 was acetylated in PTO co-expressed with HopZ3 *in planta*. Moreover, phosphorylation of S198/T199 (and T190) was reduced in the presence of HopZ3 compared to HopZ3_C300A (S2 Table and Figs 4 and S5). Since phosphorylation of S198 and T199 is necessary for immune signaling [17,22,23], this may be a part of the mechanism by which HopZ3 reduces the plant defense response to AvrPto1_*Psy*_.

### Acetylation of AvrPto1_*Psy*_ and PTO affect their binding

**Fig 6:**
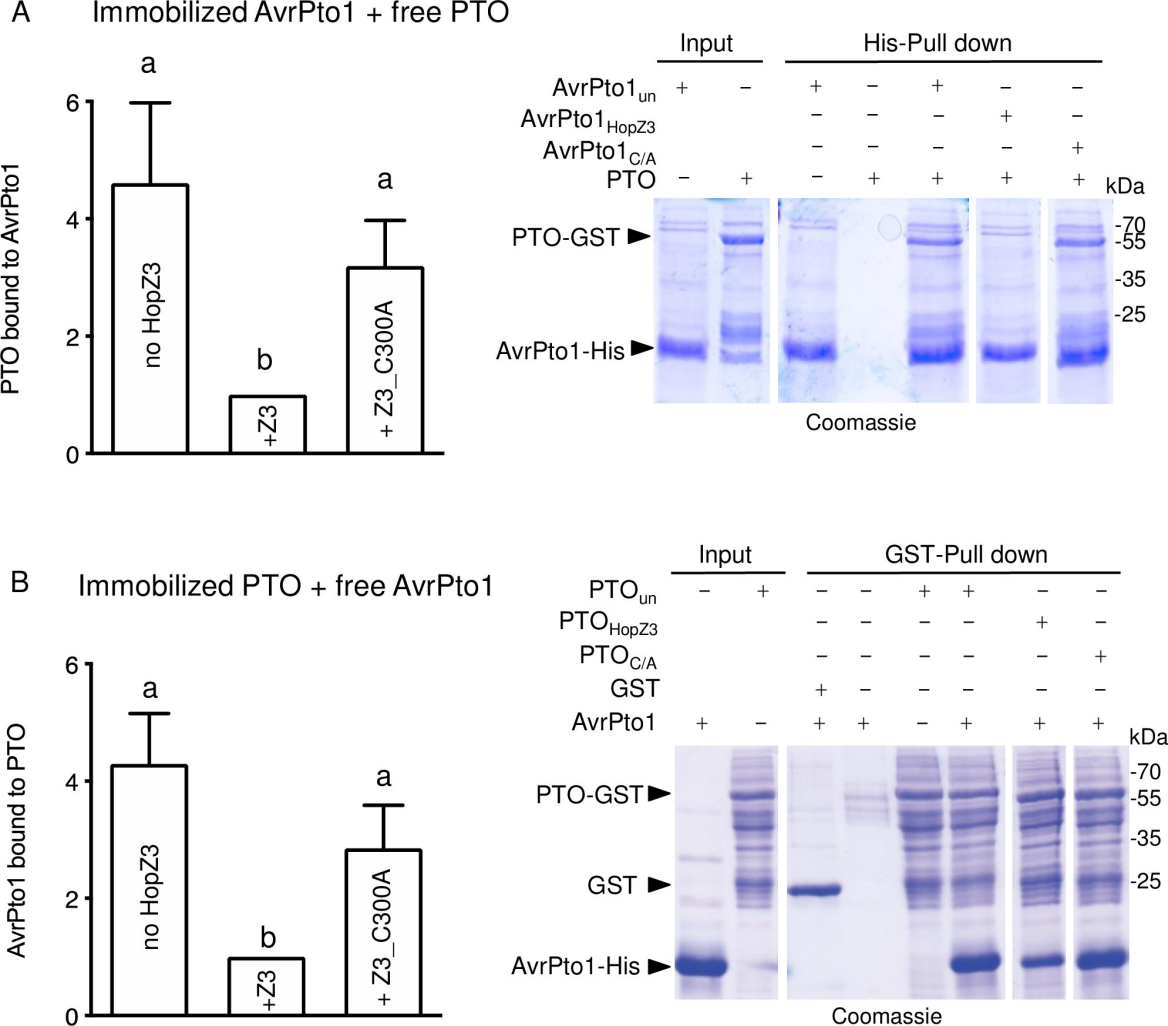
Effect of acetylation on AvrPto1_*Psy*_-PTO binding. (A) Acetylation of AvrPto1_*Psy*_ reduces its interaction with PTO. Beads with immobilized His-AvrPto1_*Psy*_ were incubated for 2 h with acetyl-CoA, IP6 and HopZ3, HopZ3_C300A (C/A), or no HopZ3 (un, untreated). Beads were washed and then incubated with soluble unmodified PTO-GST for 1 h, and after washing and elution, proteins were resolved by SDS-PAGE and stained with Coomassie blue or silver. The last two lanes in gel images are from different gels run at the same time as the other lanes, and interaction was always quantified relative to immobilized protein in the same lane. The mean with the standard error of relative band intensities from at least four experiments is shown, with binding after reaction with HopZ3 set to 1. Different letters indicate significant differences (ANOVA/Fisher’s test P<0.05). Bars indicate standard errors. (B) Acetylation of PTO reduces its binding to AvrPto1_*Psy*_. Experiments with immobilized acetylated PTO-GST and free AvrPto1_*Psy*_-His were done as in (A).

A key step in the activation of AvrPto_*Pto*_-triggered immunity requires its binding to PTO [19]. We hypothesized that modification by HopZ3 may affect the AvrPto1_*Psy*_–PTO interaction because HopZ3 targets several residues in the binding interface (Fig 4 and S1 and S2 Tables). Therefore, we assayed the impact of AvrPto1_*Psy*_ or PTO acetylation on their interaction by performing *in vitro* acetylation reactions with HopZ3 followed by binding experiments. We found that binding was reduced when either AvrPto1_*Psy*_ or PTO was acetylated (Fig 6). Thus, part of the HopZ3 mechanism of immune suppression involves inhibition of the formation of the AvrPto1_*Psy*_–PTO complex through their modification.

### Amino acid substitutions in PTO and FEN alter their acetylation specificity

**Fig 7:**
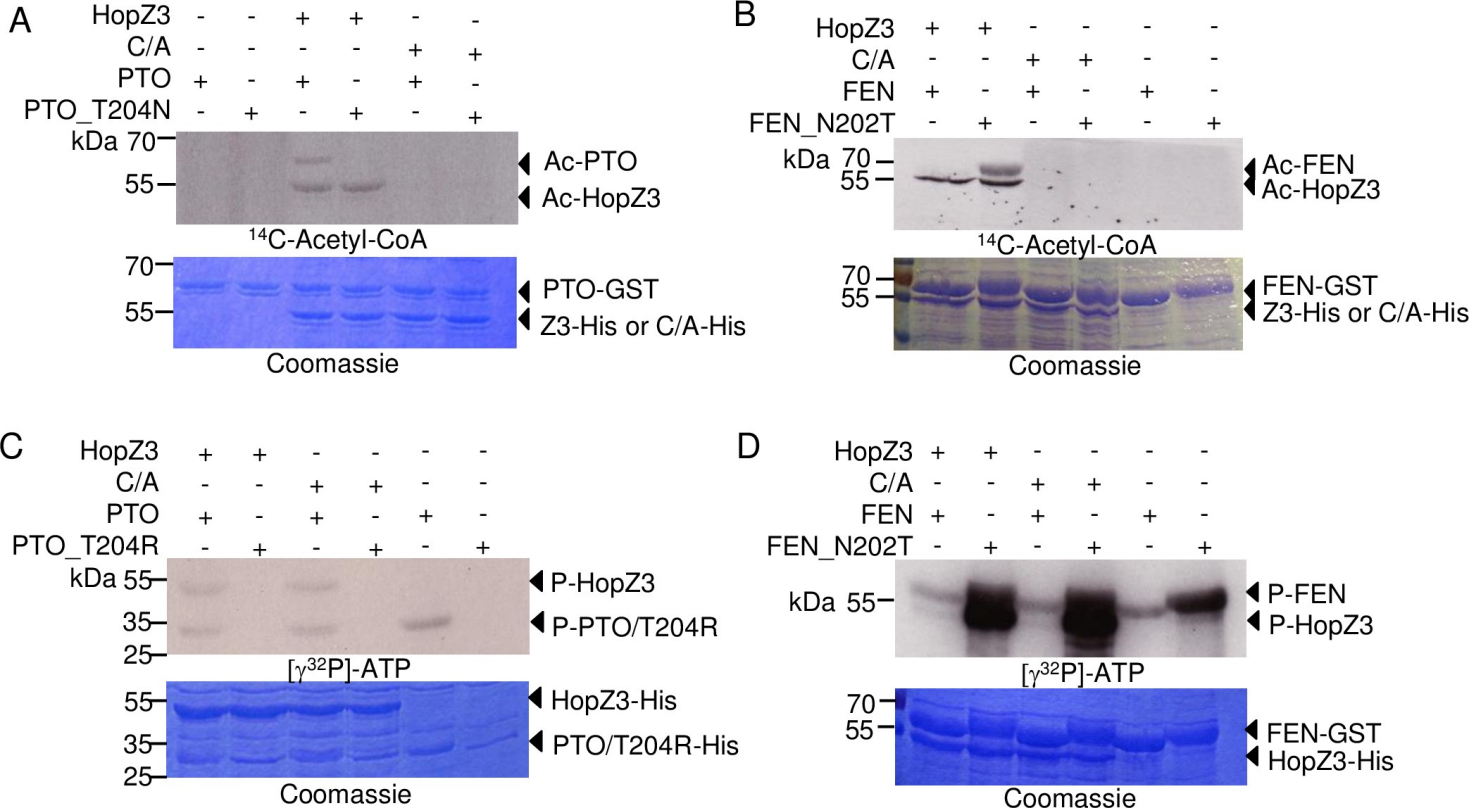
Substitutions in the P+1 activation loop of PTO and FEN affect their acetylation by HopZ3 and their kinase activity. Purified PTO-GST and FEN variants were incubated with HopZ3-His or HopZ3_C300A (C/A) mutant in the presence of IP6 and ^14^C-acetyl-CoA (A–B) or γ^32^P-ATP (C-D). Samples were separated by SDS-PAGE and subjected to autoradiography. (A) PTO but not a T204N P+1 activation loop variant was acetylated by HopZ3. (B) The substitution of Asn202 to Thr in FEN conferred acetylation by HopZ3. (C–D) Kinase activity assay showing PTO and FEN autophosphorylation and transphosphorylation of HopZ3 and HopZ_C300A *in vitro*. Kinase variants with Thr (wild-type PTO and FEN_N202T) were more active than Arg or Asn variants.

FEN has an asparagine (N202) at the cognate position to T204 in PTO. Conversion of T204 to N in PTO abolished the acetylation of the protein by HopZ3 *in vitro* (Fig 7A). Conversely, mutating N202 to T in FEN rendered it susceptible to acetylation by HopZ3 (Fig 7B). The same amino acid substitutions switched the signaling specificity of PTO and FEN in response to AvrPto_*Pto*_ as assessed by cell death induction in transient expression experiments in *N*. *benthamiana* [16]. The loss of *in vitro* acetylation of PTO_T204N by HopZ3 is consistent with our finding of only one *in vitro* acetylation site in PTO by LC-MS/MS (S2 Table).

Amino acid substitutions at position 204/202 greatly affected kinase activities of PTO and FEN, respectively. PTO and FEN variants with the T at 204/202 had higher kinase activity and showed more autophosphorylation than the N or R versions (Fig 7C and 7D; [17]). Together our data suggest that HopZ3 targets an essential residue in PTO that differentiates it from FEN in immune activation ability.

### HopZ3 acetylates multiple sites in SlRIN4s and SlRIPK

We analyzed modifications of tomato RIN4s and RIPK acetylated *in vitro* by HopZ3 using ^13^C-acetyl-CoA and found many residues to be acetylated by HopZ3 (S3 and S4 Tables). We did not observe common modified sites among all three SlRIN4 paralogues and AtRIN4; however, these proteins are not highly conserved ([9], S7 Fig). The lack of conserved acetylations may also result from the intrinsically unstructured nature of RIN4s. We found one residue that is acetylated in tomato and Arabidopsis: S88 in SlRIN4-1/S79 in AtRIN4, respectively. This residue is conserved among RIN4s from many species [9,34]. The main regulatory phosphorylation sites corresponding to AtRIN4, T166 and S141 [6] were not acetylated by HopZ3 in tomato or Arabidopsis.

The major acetylation sites in AtRIPK [9] were acetylated by HopZ3 in the tomato ortho-logue. Similar to Arabidopsis, these sites could often be also phosphorylated (S8 Fig). In particular, SlRIPK K120 (K122 in AtRIPK) in the ATP binding site, S219 (S221 in At) near the ATP binding site, SlRIPK S249/T250 (At S251/T252) in the activation loop and T255/H256 (T257 in At) were specifically acetylated by HopZ3 in both species; in addition, the serines/threonines were phosphorylation sites. K122 and S251/T252 in AtRIPK are necessary for RIPK activity [9] and S251/T252 are uridylated by the *Xanthomonas* effector AvrAC leading to RIPK inhibition [35]. Moreover, SlRIPK S249/T250 (At S251/T252) correspond to PTO S198/T199, whose phosphorylation is important for immunity [17,22,23] and is decreased by HopZ3 (S2 Table). The highest acetylation by HopZ3 was observed for SlRIPK T255, which corresponds to acetylated T257 in Arabidopsis RIPK and T204 in the PTO activation loop. Therefore, HopZ3 targets important residues conserved in SlRIPK, AtRIPK and PTO and directly acetylates SlRIPK residues necessary for kinase activity, acetylation of which may compete with phosphorylation.

### PTO, FEN and SlRIPK phosphorylate HopZ3 and SlRIN4s, and are differentially affected by HopZ3 acetylation

We tested whether kinases from the RIPK family that interact with HopZ3 can phosphorylate HopZ3 and its putative targets, SlRIN4s. Indeed, PTO, FEN and SlRIPK phosphorylated HopZ3 and SlRIN4s *in vitro* (Figs 7C, 7D and S9 and 8).

**Fig 8:**
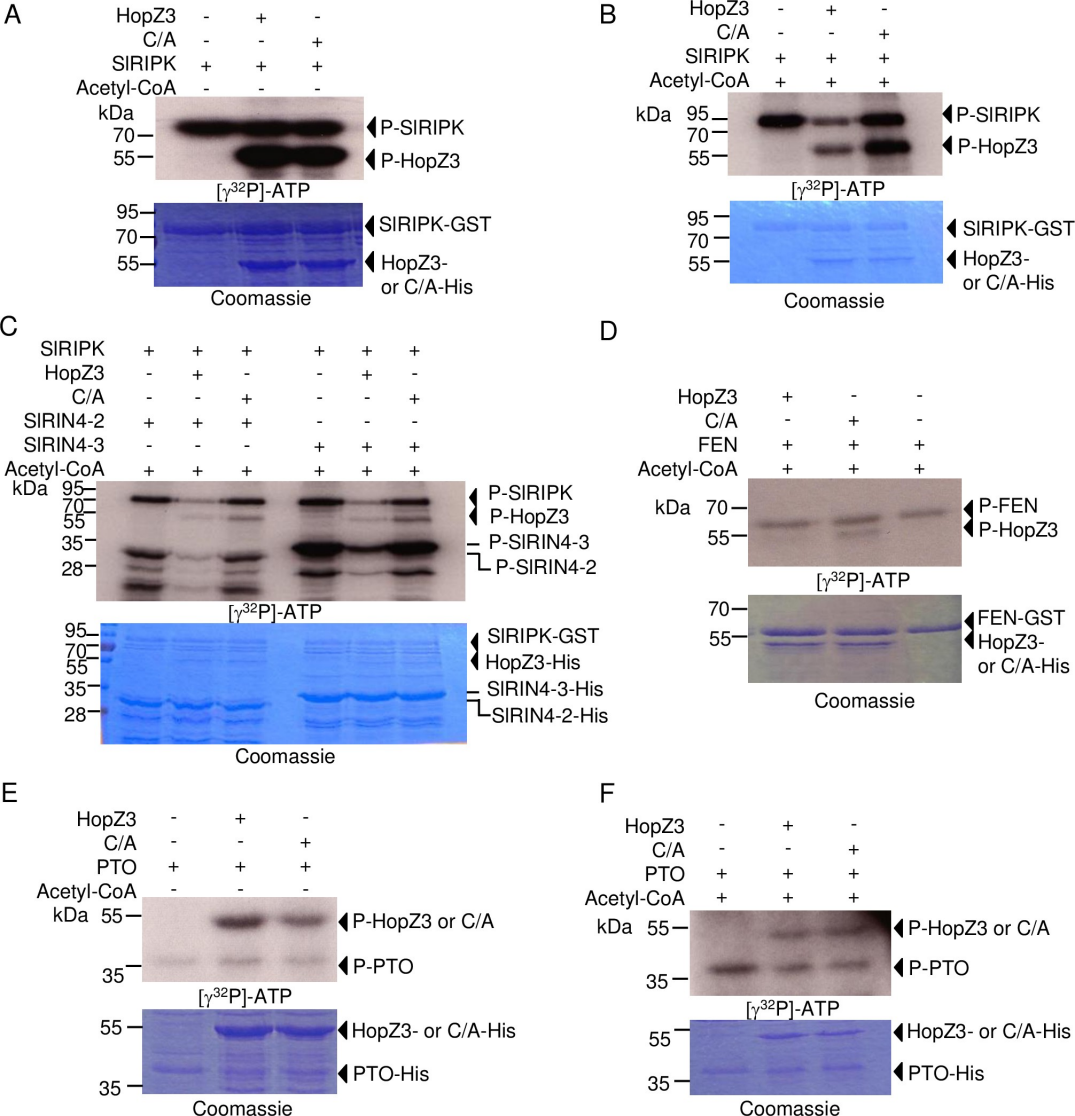
Differential effects of HopZ3 acetylation on kinase activity of RIPK, PTO and FEN. GST fusions of SlRIPK (A–C) and FEN (D) or PTO-His (E–F) were incubated with or without acetyl-CoA, IP6 and HopZ3 or HopZ3_C300A (C/A) for 2 h at RT and then washed with PBS buffer. After acetylation, the kinase activity of SlRIPK, FEN or PTO was initiated by adding γ^32^P-ATP and MgSO_4_ for 30 min. at RT. (A) Incubation of SlRIPK with HopZ3 in the absence of Acetyl-CoA did not affect the kinase activity of RIPK. (B) After incubation with acetyl-CoA and HopZ3, RIPK-mediated phosphorylation of itself and HopZ3 was reduced. (C) Phosphorylation of SlRIN4s was reduced after acetylation of SlRIPK by HopZ3. (D) Incubation with acetyl-CoA, IP6 and HopZ3 did not affect the ability of FEN to phosphorylate itself. (E–F) Acetylation of PTO by HopZ3 did not affect PTO activity.

Next, we performed acetylation reactions with HopZ3 or HopZ3_C300A followed by kinase reactions. This permitted us to test the effect of acetylation on kinase activities. Acetylation of SlRIPK greatly reduced its kinase activity and phosphorylation of SlRIN4s and HopZ3 (Fig 8A-C), similar to what we observed with Arabidopsis RIPK [9]. These results confirm that HopZ3 targets SlRIPK sites that are important for activity (S8 Fig). As expected, incubation of FEN with HopZ3 in the acetylation reaction did not affect the autophosphorylation activity of FEN (Fig 8D); however, HopZ3 phosphorylation was lower than HopZ3_C300A, possibly due to autoacetylation of HopZ3. We expected that PTO activity may be suppressed by acetylation because an R substitution at T204, the residue acetylated by HopZ3, reduced its activity (Fig 7C). However, PTO kinase activity was not strongly affected by acetylation (Fig 8E and 8F). These data show a complex network of reciprocal modifications of HopZ3 and its substrates and suggest that HopZ3 does not exert its immune-suppressing effect by direct inhibition of PTO kinase activity.

## Discussion

**Fig 9:**
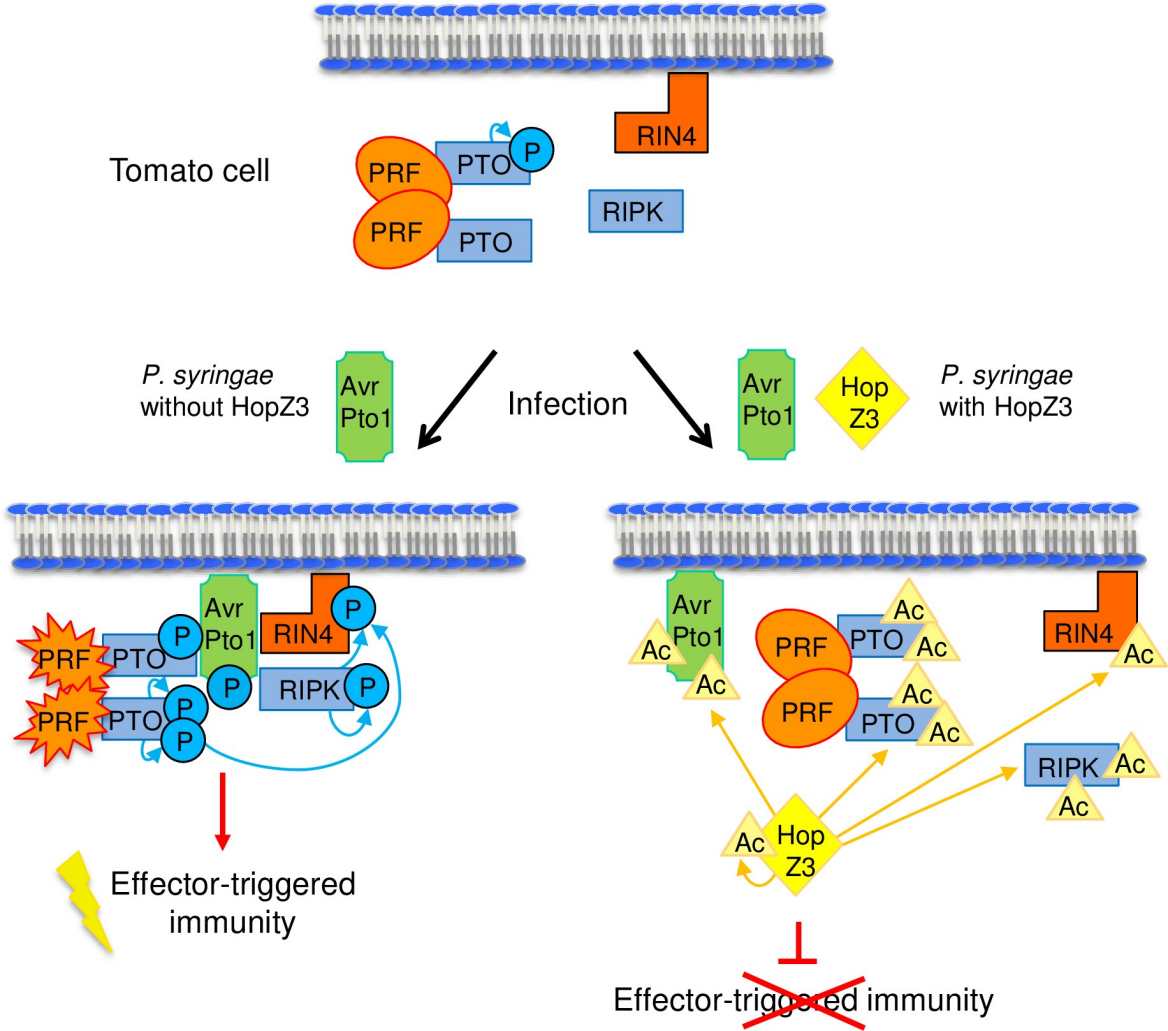
Model of HopZ3 disruption of PTO-mediated signaling in tomato. PTO and PRF are in a multimeric complex under basal conditions [23]; it is unknown whether RIN4 and/or RIPK are in a complex. AvrPto and RIN4 were shown to be associated with the membrane, but it is not known where the interactions occur. Upon infection with *P*. *syringae* containing AvrPto1_*Psy*_ but not HopZ3, AvrPto1_*Psy*_ becomes phosphorylated and binds to a PTO molecule, inhibiting its activity. Another molecule of PTO can autophosphorylate and transphosphorylate PTO bound to AvPto1_*Psy*_. The AvrPto1_*Psy*_-PTO interaction and phosphorylations cause PRF activation and initiation of effector-triggered immunity [23]. RIN4 interacts with AvrPto1_*Psy*_ and may be phosphorylated by RIPK and/or PTO during infection and contribute to signaling. In the presence of HopZ3, acetylation of AvrPto1_*Psy*_, PTO, RIPK and RIN4 leads to reduced phosphorylation and suppression of AvrPto1_*Psy*_-PTO complex formation, ultimately resulting in disruption of effector-triggered immunity.

In this study, we explored the hypothesis that the HopZ3-dependent mechanism of suppressing effector immune induction is conserved in diverse plant species, even when the effectors triggering defenses and components of the plant immune complexes are different. In resistant tomato, phosphorylation plays a prominent role in immune activation, with phosphorylated residues in effector and plant proteins promoting signaling [17,22,23,32,33]. The PTO kinase binds to the AvrPto effector, leading to the robust PRF-dependent restriction of bacterial growth. This study points to several mechanisms by which HopZ3 disrupts the PTO pathway, as outlined in the model in Fig 9. In one mechanism, acetylation of residues at the binding interface of AvrPto1_*Psy*_ (T91, S94) and PTO (T199, T204) or other residues needed for binding (S46 in AvrPto1_*Psy*_), disrupt the AvrPto1_*Psy*_–PTO interaction and subsequent immune responses. Acetylation can also directly compete at other sites for phosphorylation events that promote activity/signaling of the targets. For example, S147/S149 residues in AvrPto1_*Psy*_ and T199 in PTO are acetylated *in planta*, and phosphorylation of these residues is decreased in the presence of active HopZ3. An additional mechanism could be inactivation of kinases by acetylation; HopZ3 may also inhibit the unknown plant kinase(s) that phosphorylates AvrPto1_*Psy*_. It is also possible that acetylated AvrPto1_*Psy*_ is a poor kinase substrate. Although we did not observe *in vitro* suppression of PTO kinase activity by acetylation, it might be affected *in planta*, where more residues in the activation domain are acetylated.

In addition to acetylation at serine, lysine or threonine typically seen with YopJ family acetyltransferases, HopZ3 can also modify histidine [9]. Here we confirmed this unusual activity of HopZ3, as several histidines in AvrPto1_*Psy*_ and SlRIPK were acetylated. In AvrPto1_*Psy*_, H125/H130 residues are targets of HopZ3 acetylation and are required for the immune-inducing activity of AvrPto1_*Psy*_ in tomato. A similar observation was made in AvrB3, where substitution of H221 mitigated defense activation [9]. Although AvrPto1_*Psy*_ histidine substitution did not alter protein stability or binding to PTO, these sites might facilitate other protein dynamics or binding to different immune components.

Residues corresponding to T204, S198 and T199 in PTO were also acetylated by HopZ3 in RIPK from Arabidopsis [9] and tomato, interfering with phosphorylation and decreasing RIPK activity. Both PTO and SlRIPK (like AtRIPK [9]) could phosphorylate HopZ3 and three tomato RIN4 homologues. SlRIN4-1 is involved in PTO-PRF immunity triggered by several effectors, including AvrPto_*Pto*_ and AvrPtoB_*Pto*_, that lead to its degradation [24]. In Arabidopsis, both RIN4 degradation by AvrRpt2 and phosphorylation by RIPK triggered by AvrRpm1 and AvrB, induce immunity. This phosphorylation is prevented by HopZ3, which modifies Arabidopsis RIPK, RIN4, AvrRpm1 and AvrB3 [9]. In tomato, HopZ3 also modifies the aforementioned proteins and reduces SlRIPK activity *in vitro*, resulting in the reduced phosphorylation of SlRIN4s. The significance of the phosphorylation of SlRIN4s in tomato is unknown, but their perturbations may be guarded by R proteins and involved in immunity via a mechanism similar to that in Arabidopsis.

Tomato kinases and SlRIN4s targeted by HopZ3 also interact with AvrPto1_*Psy*_. Moreover, AvrPto1_*Psy*_, AvrPtoB_*Psy*_ and several other HopZ3 targets interact with each other. Many effectors target the same host hub proteins essential for immunity [36], including multiple kinases involved in defense [37]. Interestingly, in bean the epistatic relationship between AvrPto1_*Psy*_ and HopZ3 is reversed from that seen in tomato such that AvrPto1_*Psy*_ suppresses defenses induced by HopZ3 [38]. Epistatic interactions of the effector repertoire (effectome) are revealed in the context of the host immune repertoire (targetome) [39]. It is possible that bacterial effectors act as multi-effector anti-immune complexes, similar to plant immune complexes. Further research is needed to reveal the dynamics of these mixed plant–effector complexes. HopZ3 modification of multiple components of host defense pathways and bacterial effectors themselves may provide ways to balance the suppression of immune responses in different plants while maintaining the virulence functions of effectors.

A survey of public databases suggests that HopZ3 homologues are not present in *P*. *syringae* pv. tomato strains sequenced to date. However, many *P*. *syringae* strains contain HopZ3 and we do not know if they can infect tomato. Pathogens constantly evolve, acquire (or lose) new effectors and this may enable infection of new plant species. It is plausible that tomato pathovars could acquire HopZ3 and overcome PTO/PRF-mediated disease resistance in the future, or a HopZ3-containing strain could become adapted to tomato. Epistatic interactions between effectors determine host range and effector loss and gain allow changes in host range.

Remarkably, some of HopZ3 immune modulations mirror those of other YopJ family acetyltransferases. Effectors in human and animal pathogens, such as YopJ in *Yersinia* sp., AvrA in *Salmonella* and VopA in *Vibrio*, acetylate residues in activation loops and ATP binding sites of kinases in MAPK and IKK pathways, blocking their phosphorylation [40]. Plant pathogen YopJ family effectors from *Pseudomonas*, *Ralstonia* and *Xanthomonas* are much more diverse and are known to have a large spectrum of unrelated substrates [40]. So far, HopZ3 is unique in its strategy to modify other bacterial effectors in addition to their plant targets to reduce immune responses. The ability to post-translationally modify its own effectors adds another layer to the bacterial arsenal, in addition to the acquisition of effectors suppressing PAMP- or effector-triggered defenses and the evolution of multiple effector alleles that can avoid recognition.

## Material and methods

### Plant growth and bacterial infection

Tomato (*Solanum lycopersicum*) plants had the Rio Grande-PtoR (76R) background that has *Pto/Prf* locus introgressed from resistant *S*. *pimpinellifolium*; *pto11* and *prf3* are lines with mutated, nonfunctional *Pto* and *Prf* genes, respectively [41]. Tomato and *Nicotiana benthamiana* plants were grown under standard greenhouse conditions (22–24°C and 16/8 h light/dark photoperiods). Bacterial infection with *P*. *syringae* pv. *syringae* strain *Psy*B728a (and derivatives thereof) was performed with 4-week-old plants. Tomato plants were sprayed with a bacterial suspension (O.D _600_ = 0.01 with 0.02% Silwet in 10 mM MgSO_4_) and covered with a dome without holes. Eight to twelve leaf discs from at least four infected plants were collected 3–5 days post inoculation. For total bacteria count, individual discs were homogenized in 200 μl of 10 mM MgSO_4_ and for epiphytic bacteria count, discs were washed to detach surface bacteria by vortexing in 1 ml of 10 mM MgSO_4_ [7,8]. Samples were serially diluted and plated on LB medium containing appropriate antibiotics. Bacterial growth experiments were performed at least three times. Results obtained with total and epiphytic bacteria counts were very similar and these experiments were used interchangeably. Transient transformation of *N*. *benthamiana* leaves using Agrobacterium was performed as previously described [7]. Bacterial strains are listed in S5 Table.

### Plasmid construction

For Gateway cloning vectors, the open reading frame (ORF) of each gene was amplified without a stop codon using Pfu-DNA polymerase (Agilent Technologies) and the entire region was cloned into pDONR207 by Gateway BP reaction (Life Technologies) and then recombined by Gateway LR reaction (Life Technologies) into the destination vectors (pG005/pG006 for BiFC, pLaw vectors for yeast two-hybrid assay, pBAV226 for expression in *Psy*B728a). Point mutations were introduced by PCR using overlapping primers with mutated codons. The *E*. *coli* protein expression vectors used in this study (S6 Table) are not Gateway compatible. The ORFs were amplified using gene-specific primers with restriction enzyme sites at the 5’-end or 3’-ends. PCR products were digested with specific restriction enzymes and ligated into expression vectors. All constructs were verified by sequencing. Details of primers, vectors, bacterial and yeast strains are provided in S5–S7 Tables.

### Effector deletion strains and complementation

Unmarked deletions of AvrPto1 in *Psy*B728a and *Psy*B728aΔHopZ3 [8] were created as described [8,9]. Briefly, regions upstream and downstream of AvrPto1were amplified with 5’and 3’primers (S7 Table) and linked together in pMTN1907 that has SacB cassette for negative selection. Colonies with integrated plasmid were selected on kanamycin, and subsequently deletion strains were selected on 10% sucrose. Deletion strains were complemented with effectors expressed from the *nptII* promoter in the low-copy pBAV226 plasmid as previously described [8]. Details of vectors and primers are provided in S6 and S7 Tables.

### GenBank accession numbers

GenBank accession numbers of proteins used in this study: AvrPto1_*Psy*_: AAY39946; AvrPtoB_*Psy*_ (HopAB1_*Psy*_): Q4ZMD6; PTO: AAB47423; FEN: AAB47424; SlRIPK: AAK62821; AtRIPK: NP_178651; SlRIN4-1: XP_010326285; SlRIN4-2: XP_004242410; SlRIN4-3: XP_004252989; AtRIN4: NP_189143.

### Yeast two-hybrid assay

The yeast two-hybrid screen was a part of a large scale effector-plant immune signaling protein interaction screen ([9], https://charge.ucdavis.edu/charge_db/interaction/Y2H/Y2H_interaction.php), and identified interactions were confirmed as previously described [9]. Briefly, the corresponding cells of the bait and prey were mated as shown in S1 Fig. Mated yeast strains (S5 Table) expressing the bait and prey constructs were grown on the selective minimal SD media (SD-Leu/-Trp/-His supplemented with 2.0 mM 3-aminotriazole (3-AT and SD-Leu/-Trp/+X-gal) for 4–6 days. Experiments were performed at least twice.

### *In vitro* pull-down assay

*In vitro* pull-down assays were performed between purified recombinant GST-tagged SlPTO, -SlFEN, -SlRIN4-2, -3 or SlRIN4-1-MBP and His-tagged HopZ3; between His-tagged AvrPto1_*Psy*_ or AvrPtoB_*Psy*_ and GST-tagged HopZ3, -PTO, or -FEN or PTO-MBP as described [9]. Mixed proteins were incubated at 4°C for 1–2 h. Protein bound to the glutathione-sepharose beads (GE Healthcare or Promega), Ni-NTA agarose (QIAGEN) or amylose beads (NE BioLabs) was washed three to four times, separated on SDS-PAGE and stained with Coomassie blue or immunoblotted with anti-GST, anti-MBP and anti-His antibodies, respectively. All experiments were performed at least twice.

To assess a protein–protein interaction after acetylation by HopZ3, beads with immobilized AvrPto1_*Psy*_-His or PTO-GST were incubated with 1 mM Acetyl-CoA, 5 μM IP6 and 1 μg HopZ3 or HopZ3_C300A for 2 h at room temperature (RT), washed three times, then the second interacting protein was added and pull down was performed as described above. Relative band intensities (interacting protein relative to immobilized protein) were quantified from at least four independent experiments using Image Lab software (Bio-Rad). To compare different experiments, interaction after acetylation with HopZ3 was set to 1.

### Immunoblotting

Proteins were resolved by 12% SDS-PAGE, transferred to a PVDF membrane and probed with α-GST (Biolegend), α-His_6_ (Clontech), α-MBP (NE BioLabs), α-GFP (Clontech) and α-HA (Covance) antibodies followed by HRP-fused secondary antibodies (Thermo Fisher Scientific). Blots were developed with chemiluminescent SuperSignal Pico West solution (Thermo Fisher Scientific).

### BIFC assay and confocal microscopy

For BIFC analysis, protein-coding sequences were cloned into expression plasmids pG005 to create protein fused to the N-terminal half of YFP (protein:nYFP fusions) and into pG006 to create protein fused to the C-terminal half of YFP (protein:cYFP fusions), as previously described [9]. *N*. *benthamiana* leaves were co-infiltrated with mixtures of Agrobacteria harboring indicated combinations of BIFC constructs and YFP fluorescence was imaged 2 days after agroinfiltration. A LSM710 confocal laser scanning microscope (Zeiss Microsystems) equipped with a 40X water-immersion objective was used to examine protein subcellular localization or protein–protein interaction in BIFC assays with *N*. *benthamiana* epidermal cells. GFP or YFP imaging was performed by excitation with 488 nm argon laser and emission at 496–544 nm for GFP and 494–573 nm for YFP. YFP fluorescence indicated interaction. Experiments were repeated two to three times.

### *In vitro* acetylation

Purified His-tagged HopZ3 or -HopZ3_C300A (0.5–1 μg) and 1–5 μg of potential substrates (GST tagged PTO, PTO_T204N, FEN and FEN_N202T; His-tagged SlRIN4-1, SlRIN4-2, SlRIN4-3, AvrPto1_*Psy*_ and AvrPtoB_*Psy*_) were incubated with an acetylation reaction mixture containing 50 mM HEPES (pH 8.0), 10% glycerol, 5 μM IP6 and 1–2 μl ^14^C-acetyl-coenzyme A (56 μCi/μM) (PerkinElmer Life Science) in a total volume of 20 μl as previously described [9]. The reactions were incubated for 2 h at RT and were terminated by the addition of SDS-PAGE loading buffer and boiling for 5 min. Proteins were separated by 12% or 15% SDS-PAGE, gels were dried on 3M paper and exposed to X-ray film for 7–14 days at -80°C. Experiments were performed two to three times.

### *In vitro* kinase assay

*In vitro* kinase assays were performed as previously described [9]. Briefly, 0.2, 0.4 and 0.6 μg of purified GST-tagged PTO or -FEN or 0.5 μg of purified GST-tagged SlRIPK were incubated with a buffer containing 100 mM Tris 6.8, 10 mM MgCl_2_, 10 mM MnCl_2_, 10 μM ATP and 1 μl of γ-^32^P-ATP and adding 2 μg of His-tagged SlRIN4-1 or HopZ3 at RT for 60 min. The reaction was stopped by adding 5x Laemmli buffer. Proteins were separated by 12 or 15% SDS-PAGE, and signals were visualized by autoradiography.

To determine kinase activity after acetylation by HopZ3, 1 μg of SlRIPK-GST, FEN-GST or PTO-His were incubated with 1 mM Acetyl-CoA, 5 μM IP6 and 1 μg of His-Tagged HopZ3 or HopZ3_C300A for 2 h at RT and then washed with PBS. The kinase activity of SlRIPK, FEN or PTO was initiated by adding ATP, γ-^32^P-ATP and MgSO_4_ with or without SlRIN4s and incubated for 30 min at RT. All experiments were performed two to three times.

### *In vitro* PTM mapping

For *in vitro* acetylation mapping, reactions were performed with ^13^C-acetyl-CoA (Acetyl-1,2-^13^C coenzyme A lithium salt, Sigma-Aldrich) to differentiate between background ^12^C-acetylation that occurred in *E*. *coli* during the synthesis of recombinant protein and HopZ3-mediated acetylation *in vitro*. Substrates were mixed with either HopZ3 or the catalytically inactive HopZ3_C300A to distinguish any background acetylation that could be chemically caused by the presence of ^13^C-acetyl-CoA. Briefly, 1 μg of purified His-tagged HopZ3 or HopZ3_C300A were mixed with 3 μg bead-bound substrate to which the acetylation reaction cocktail (50 mM HEPES (pH 8.0), 10% glycerol, 5 μM IP6 and 50 μM of ^13^C-acetyl-CoA (Sigma-Aldrich)) was added in a total volume of 20 μl. Subsequently, beads were washed twice with washing buffer (50 mM HEPES pH 8.0, 50 mM NaCl, 10% glycerol), boiled in Laemmli loading buffer and processed for LC-MS/MS analysis. Data from the mass spectrometry of treated samples were analyzed for the presence of ^13^C-acetylated peptides in the substrate (AvrPto1_*Psy*_, PTO, SlRIN4s, SlRIPK).

### Immunoprecipitation and *in planta* PTM mapping

For *in planta* acetylation mapping, Dex-AvrPto1_*Psy*_-HA or Dex-PTO-HA were transiently co-expressed with Dex-HopZ3-GFP or Dex-HopZ3_C300A-GFP constructs in *N*. *benthamiana*. Plants were treated with 30 μM dexamethasone for 16 h to induce protein production. Proteins were extracted with lysis buffer containing 50 mM Tris pH 8.0, 150 mM NaCl, 10% glycerol, 1% NP40, 0.5% sodium deoxycholate, phosphatase inhibitor (Thermo Fisher Scientific), 2 μM sodium butyrate (TOCRIS Bioscience) and 3 μM trichostatin A (Sigma-Aldrich). Clarified total protein lysate was incubated for 3 h with anti-HA magnetic beads (Medical and Biological Laboratories Co., LTD) at 4°C. After washing the beads three times with the lysis buffer, proteins were eluted by boiling with Laemmli loading buffer. Samples were analyzed by LC-MS/MS. PTM mapping of AvrPto1_*Psy*_ and PTO was repeated with independent experiments.

### LC-MS/MS analysis

Trypsin digestion and HPLC were performed as described [9]. Mass spectrometry was performed at the Medical Genome Facility Proteomics Core at Mayo Clinic, Rochester, MN, US. Samples were analyzed via liquid chromatography-electrospray tandem mass spectrometry (LC-MS/MS) on a Q-Exactive (Thermo Fisher Scientific) mass spectrometer, using a 60,000 RP survey scan, *m/z* 375–1950, with lockmasses, followed by 15 HCD (higher energy collisional dissociation) CID (collision-induced dissociation) scans on only doubly and triply charged precursors between 375 and 1950 Da and ions selected for MS/MS were placed on an exclusion list for 60 seconds. Inclusion lists were applied to enhance the detection of acetylated or phosphorylated peptides from specific targets. Briefly, using in house software to process the FASTA sequence file for AvrPto1_*Psy*_, PTO, tomato RIN4_1–3 and SlRIPK, we performed *in silico* trypsinization and modeled the following modifications: (formyl n-term, oxidation (M), acetyl (K, H, S, T), ^13^C heavy acetyl (K, H, S, T), phospho (S, T), myristoylation (N-terminal G)), calculated *m/z* for doubly and triply charged ions, and combined the results into a *.csv file that was applied to the QE instrumentation method to enhance selection of the PTM-bearing ions for fragmentation. The MS data have been deposited to the ProteomeXchange Consortium (http://proteomecentral.proteomexchange.org) with the dataset identifier PXD022953. Database searching of the 160610_Greenberg_db9 database (3412 entries) and protein identification and PTM quantification were performed as described in [9] and [42]. All acetylated, phosphorylated and myristoylated peptide spectra were manually validated [9]. The second *in planta* experiment was quantified by TIC (total ion current) using Scaffold [43]. PTMs above 5% are shown in S1–S4 Tables.

### Structural modeling

To assess the relevance of the acetylated residues found by mass spectrometry, we modeled the structure of the HopZ3 substrates using the iTASSER (Iterative Threading Assembly Refinement) structural prediction software as previously described [9]. The best possible model was selected based on confidence score (C-score) calculated based on the significance of threading to the template alignments and convergence to the parameters of the structural assembly simulations. Model visualizations were generated using PyMOL software. PTM and interaction sites were labeled using the stick setting and coloring (Fig 4); however, the sites in the model are shown without PTMs.

## Supporting information

S1 Fig Yeast two-hybrid assay. Positive interactions are indicated by the growth on the selection medium without Trp, Leu and His (SD-WLH+5mM 3-AT) for the reporter gene HIS3 or by blue color on medium containing X-gal [9]. A schematic overview of a subset of tested combinations is represented in Table 1. SlRIN4-3_trunc_ was used as a negative control; it has a deletion of nucleotide 14 that caused a frameshift mutation and early stop. FEN as a bait caused auto-activation (false positive).
(PDF)

S2 Fig Interaction between HopZ3, AvrPto1_*Psy*_ and their potential interactors *in planta*. Interactions of HopZ3, AvrPto1_*Psy*_, AvrPtoB_*Psy*_, PTO, FEN, SlRIPK, SlRIN4-1, -2 and -3 were tested by BiFC. YFP fluorescence was imaged by confocal microscopy in epidermal *N*. *benthamiana* cells co-infiltrated with mixtures of Agrobacteria harboring expression plasmids pG005 (protein:nYFP fusions) and pG006 (protein:cYFP fusions). Bar = 20 μm. Schematic overview of a subset of tested combinations is represented in Table 1: +, fluorescence detected; -, fluorescence not detected; weak, weak signal, as determined from images of several experimental samples.
(PDF)

S3 Fig Pull downs with recombinant tagged proteins to assess interaction between AvrPto1_*Psy*_ and proteins in the PTO immune pathway. Empty Ni^2+^ resin or immobilized GST were used as negative controls for His- and GST- pull downs, respectively. Proteins were detected by Coomassie staining or immunoblotting. (A) Immobilized His-tagged AvrPto1_*Psy*_ pulled down purified PTO-MBP. (B) Immobilized AvrPto1_*Psy*_ -His was incubated with FEN-GST, showing no interaction. (C) Immobilized His-tagged AvrPto1_*Psy*_ was incubated with SlRIN4-1-MBP showing weak interaction in one experiment and no interaction in two experiments. (D) Immobilized His-tagged AvrPto1_*Psy*_ was incubated with SlRIN4-2-GST showing weak interaction in two of three experiments. (E) Immobilized His-tagged AvrPto1_*Psy*_ was incubated with SlRIN4-3-GST. Interaction was not detected. (F) Immobilized GST-AvrPto1_*Psy*_ or GST was mixed with His-tagged AvrPtoB_*Psy*_, showing that the two effectors interact.
(PDF)

S4 Fig HopZ3 acetylates multiple sites in AvrPto1_*Psy*_ important for interaction with PTO and signaling. MS/MS spectra show PTM of AvrPto1_*Psy*_ expressed in *N*. *benthamiana* in the presence of HopZ3 or HopZ3_C300A. (A) Evidence of G2 myristoylation. (B) Acetylation of H125 and H130 in the presence of HopZ3. S136 was phosphorylated in both samples (HopZ3 and HopZ3_C300A). (C) Acetylation of T91 and S94 observed in the presence of HopZ3. (D–F) Acetylation of S147 and S149 in the presence of HopZ3 (D) reduced phosphorylation of these residues. Phosphorylation was observed in the presence of HopZ3_C300A (E–F).
(PDF)

S5 Fig HopZ3 acetylates key sites in the activation loop of PTO. PTMs were analyzed using mass spectrometry for PTO after *in vitro* acetylation reaction (A) or co-expressed with HopZ3 or HopZ3_C300A in *N*. *benthamiana* (B–E). (A–B) In both *in vitro* (A) and *in planta* (B) analyses T204, a key residue in the activation loop of the PTO kinase, was acetylated in the presence of HopZ3. (C) T199 acetylation in the presence of HopZ3. (D–E) Phosphorylation of S198 and T199 in the presence of HopZ3_C300A.
(PDF)

S6 Fig Effect of mutations of AvrPto1_*Psy*_ acetylation sites on cell death induction in *N*. *benthamiana*. AvrPto1_*Psy*_-GFP variants were transiently expressed in *N*. *benthamiana* infiltrated with Agrobacterium at OD = 0.2 or 0.4 and sprayed with dexamethasone. (A) Only AvrPto1_*Psy*__T91A induced delayed cell death compared to wild-type AvrPto1_*Psy*_. All variants were expressed to similar levels. A number of infiltrated areas with cell death per total number of samples is shown in the tables. (B) H125, H130 and double mutant of AvrPto1_*Psy*_ induced cell death in *N*. *benthamiana*.
(PDF)

S7 Fig Alignment of tomato RIN4s (4_1, 4_2, 4_3) and Arabidopsis RIN4 (AT). Modifications in SlRIN4s were determined *in vitro*, modifications in AtRIN4 are from [9] (*in vitro* and *in planta*). Residues acetylated by HopZ3 are bold and highlighted in yellow; phosphorylation sites are underlined; known phosphorylation sites important for signaling (S141, T166) in AtRIN4 [6] are highlighted blue; residues phosphorylated by RIPK in AtRIN4 (T21, S160, T166) [3] are circled in red. * (asterisk)—fully conserved residues,: (colon)—conservation between groups of strongly similar properties,. (period)—conservation between groups of weakly similar properties.
(PDF)

S8 Fig HopZ3 acetylates SlRIPK residues important for activity. Modifications in SlRIPK were determined *in vitro*, modifications in AtRIPK are from [9] (*in vitro* and *in planta*). Residues acetylated by HopZ3 are bold and highlighted in yellow; phosphorylation sites are underlined; known sites in AtRIPK important for activity (K122; S251/T252 which correspond to S198/T199 in PTO) [9] are circled in red; sites corresponding to T204 in PTO are circled in blue. * (asterisk)—fully conserved residues,: (colon)—conservation between groups of strongly similar properties,. (period)—conservation between groups of weakly similar properties.
(PDF)

S9 Fig Kinases in the PTO family can phosphorylate tomato RIN4s. (A) PTO and FEN phosphorylate tomato SlRIN4-1. Kinase assays showing an increasing amount of PTO and FEN autophosphorylation and transphosphorylation of SlRIN4-1. (B-C) SlRIPK and FEN phosphorylate SlRIN4-3. The time course of the *in vitro* kinase reactions is shown. Purified SlRIPK (B) or FEN (C) were incubated in kinase buffer with or without SlRIN4-3 as a substrate. At indicated time points, aliquots of the reaction were taken out and separated by SDS-PAGE.
(PDF)

S1 Table AvrPto1_*Psy*_ PTMs *in vitro* and *in planta*. PTMs were determined either *in vitro*, using purified recombinant AvrPto1_*Psy*_ after ^13^C-acetylation by HopZ3/HopZ3_C300A, or *in planta*, by co-expressing AvrPto1_*Psy*_ and HopZ3/HopZ3_C300A in *N*. *benthamiana*, followed by immunoprecipitation. Numbers indicate enrichment (fold change) of acetylation in the presence of HopZ3 vs. HopZ3_C300A. Red shading: significant (>50%) increase of acetylation with HopZ3. Blue shading: significant decrease of phosphorylation *in planta* in the presence of HopZ3. Residues known to be important for AvrPto signaling or interaction with PTO are in bold. + indicates phosphorylation found in a recombinant protein (*in vitro*) or *in planta*. Z3: acetylation found only in AvrPto1_*Psy*_ treated or co-expressed with HopZ3 and not HopZ3_C300A. Ac: acetylation; Phos: phosphorylation; Myr: myristoylation; exp: experiment. *Some spectra do not distinguish these 2 close residues. ^#^*In planta* sites with acetylation above 25% in the presence of HopZ3.
(PDF)

S2 Table PTO PTMs *in vitro* and *in planta*. PTMs were determined either *in vitro*, using purified recombinant PTO after ^13^C-acetylation by HopZ3/HopZ3_C300A, or *in planta*, by co-expressing PTO and HopZ3/HopZ3_C300A in *N*. *benthamiana*, followed by immunoprecipitation. Numbers indicate enrichment (fold change) of acetylation in the presence of HopZ3 vs. HopZ3_C300A. Red shading: significant (>50%) increase of acetylation with HopZ3. Blue shading: significant decrease of phosphorylation *in planta* in the presence of HopZ3. Residues important for PTO signaling or interaction with AvrPto are in bold. + indicates phosphorylation found in a recombinant protein (*in vitro*) or *in planta*. Z3: acetylation found only in PTO treated or co-expressed with HopZ3 and not HopZ33_C300A.; Ac: acetylation; Phos: phosphorylation; exp: experiment. *Some spectra do not distinguish these 2 close residues. ^#^*In planta* sites with acetylation above 25% in the presence of HopZ3.
(PDF)

S3 Table SlRIN4s PTMs *in vitro*. PTMs were determined using purified recombinant SlRIN4s after *in vitro* ^13^C-acetylation by HopZ3/HopZ3_C300A. Numbers indicate enrichment (fold change) of ^13^C-acetylation in the presence of HopZ3 vs. HopZ3_C300A. Z3: acetylation found only in SlRIN4 treated with HopZ3. Red shading: significant (>50%) increase of modification with HopZ3. + indicates phosphorylation found in a recombinant protein. Ac: acetylation; Phos: phosphorylation.
(PDF)

S4 Table SlRIPK PTMs *in vitro*. PTMs were determined using purified recombinant SlRIPK after *in vitro* ^13^C-acetylation by HopZ3/HopZ3_C300A. Numbers indicate enrichment (fold change) of ^13^C-acetylation in the presence of HopZ3 vs. HopZ3_C300A. Z3: acetylation found only in SlRIPK treated with HopZ3. Red shading: significant (>50%) increase of modification with HopZ3. + indicates phosphorylation found in a recombinant protein. Ac: acetylation; Phos: phosphorylation.
(PDF)

S5 Table Bacterial and yeast strains. (PDF)

S6 Table Plasmid vectors. (PDF)

S7 Table PCR primer sequences used in this study. Mutated codons are underlined.
(PDF)
