# Supplementary material for: Pseudomonas syringae effector HopZ3 suppresses the bacterial AvrPto1–tomato PTO immune complex via acetylation
Source: PLoS Pathog. 2021 Nov 1;17(11):e1010017. doi: 10.1371/journal.ppat.1010017 (PMC8584673; doi:10.1371/journal.ppat.1010017)
Supplement: S1 Fig — Positive interactions are indicated by the growth on the selection medium without Trp, Leu and His (SD-WLH+5mM 3-AT) for the reporter gene HIS3 or by blue color on medium containing X-gal [9]. A schematic overview of a subset of tested combinations is represented in Table 1. SlRIN4-3trunc was used as a negative control; it has a deletion of nucleotide 14 that caused a frameshift mutation and early stop. FEN as a bait caused auto-activation (false positive). (PDF) [file ppat.1010017.s001.pdf]

	Bait	Prey	interaction
1.	HopZ3	PTO	-
2.	HopZ3	FEN	+ (weak)
3.	HopZ3	SlRIPK	-
4.	HopZ3	SlRIN4-1	-
5.	HopZ3	SlRIN4-2	-
6.	HopZ3	SlRIN4-3$_{trunc}$	-
7.	PTO	HopZ3	-
8.	FEN	HopZ3	auto activation
9.	SlRIPK	HopZ3	+ (weak)
10.	SlRIN4-1	HopZ3	+
11.	SlRIN4-2	HopZ3	+
12.	SlRIN4-3$_{trunc}$	HopZ3	-

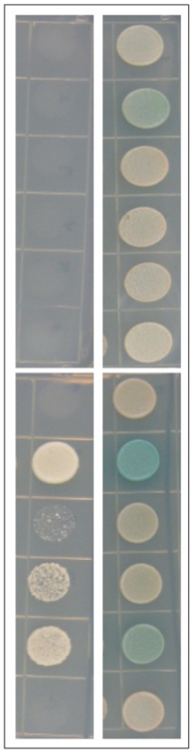

	Bait	Prey	interaction
25.	PTO	PTO	-
26.	FEN	FEN	auto activation
27.	SlRIPK	SlRIPK	-
28.	SlRIN4-1	SlRIN4-1	+ (weak)
29.	SlRIN4-2	SlRIN4-2	-
30.	SlRIN4-3$_{trunc}$	SlRIN4-3$_{trunc}$	-
31.	PTO	SlRIN4-1	-
32.	PTO	SlRIN4-2	-
33.	PTO	SlRIN4-3$_{trunc}$	-
34.	FEN	SlRIN4-1	auto activation
35.	FEN	SlRIN4-2	auto activation
36.	FEN	SlRIN4-3$_{trunc}$	auto activation

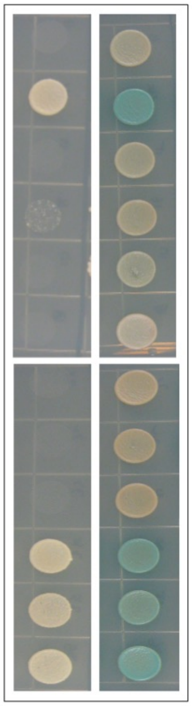

	Bait	Prey	interaction
13.	AvrPto1	PTO	+ (weak)
14.	AvrPto1	FEN	+
15.	AvrPto1	SlRIPK	+
16.	AvrPto1	SlRIN4-1	-
17.	AvrPto1	SlRIN4-2	-
18.	AvrPto1	SlRIN4-3$_{trunc}$	-
19.	PTO	AvrPto1	+
20.	FEN	AvrPto1	auto activation
21.	SlRIPK	AvrPto1	-
22.	SlRIN4-1	AvrPto1	+ (weak)
23.	SlRIN4-2	AvrPto1	+ (weak)
24.	SlRIN4-3$_{trunc}$	AvrPto1	-

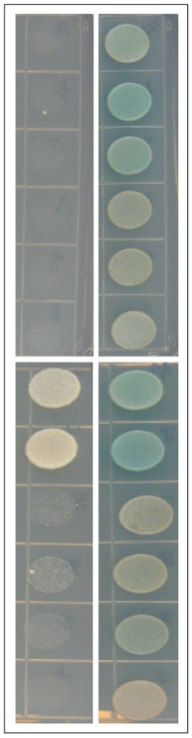

	Bait	Prey	interacti
37.	SlRIPK	SlRIN4-1	-
38.	SlRIPK	SlRIN4-2	-
39.	SlRIPK	SlRIN4-3$_{trunc}$	-
40.	SlRIN4-1	PTO	-
41.	SlRIN4-2	PTO	+ (weak)
42.	SlRIN4-3$_{trunc}$	PTO	-
43.	SlRIN4-1	FEN	+ (weak)
44.	SlRIN4-2	FEN	+
45.	SlRIN4-3$_{trunc}$	FEN	-
46.	SlRIN4-1	SlRIPK	+ (weak)
47.	SlRIN4-2	SlRIPK	+
48.	SlRIN4-3$_{trunc}$	SlRIPK	-

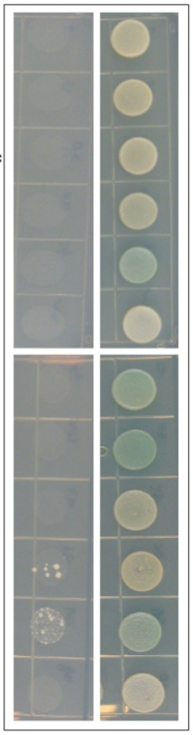

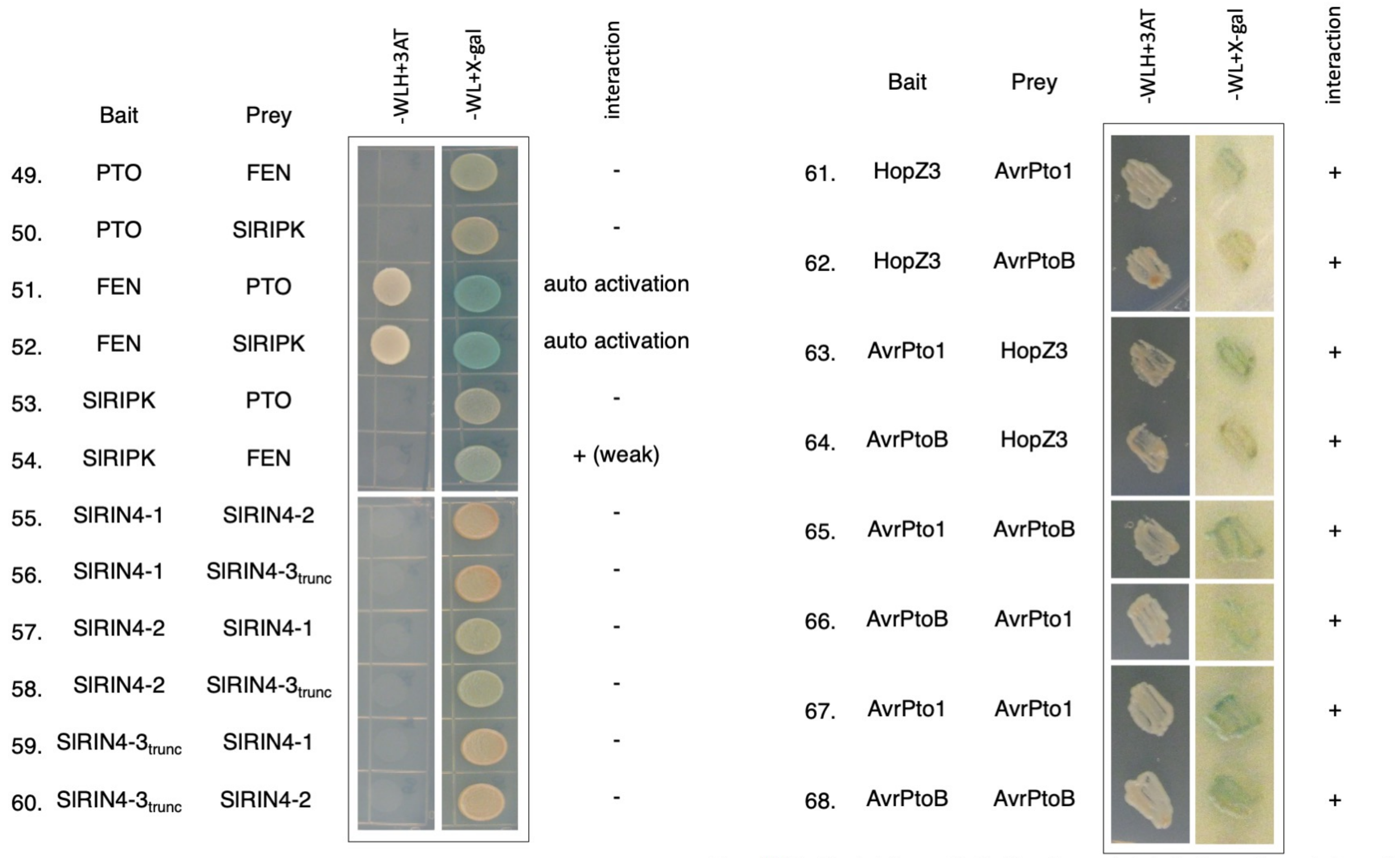

S1 Fig. Yeast two-hybrid assay. Positive interactions are indicated by the growth on the selection medium without Trp, Leu and His (SD-WLH+5mM 3-AT) for the reporter gene HIS3 or by blue color on medium containing X-gal [1]. A schematic overview of a subset of tested combinations is represented in Table 1. SlRIN4-3$_{trunc}$ was used as a negative control, it has a deletion of nucleotide 14 that caused a frameshift mutation and early stop. FEN as a bait caused auto-activation (false positive).

References

1. Lee J, Manning AJ, Wolfgeher D, Jelenska J, Cavanaugh KA, Xu H, et al. Acetylation of an NB-LRR plant immune-effector complex suppresses immunity. Cell Rep. 2015;13(8):1670-82. doi: 10.1016/j.celrep.2015.10.029. PubMed PMID: 26586425.
