# Supplementary material for: Pseudomonas syringae effector HopZ3 suppresses the bacterial AvrPto1–tomato PTO immune complex via acetylation
Source: PLoS Pathog. 2021 Nov 1;17(11):e1010017. doi: 10.1371/journal.ppat.1010017 (PMC8584673; doi:10.1371/journal.ppat.1010017)
Supplement: S2 Fig — Interactions of HopZ3, AvrPto1Psy, AvrPtoBPsy, PTO, FEN, SlRIPK, SlRIN4-1, -2 and -3 were tested by BiFC. YFP fluorescence was imaged by confocal microscopy in epidermal N. benthamiana cells co-infiltrated with mixtures of Agrobacteria harboring expression plasmids pG005 (protein:nYFP fusions) and pG006 (protein:cYFP fusions). Bar = 20 μm. Schematic overview of a subset of tested combinations is represented in Table 1: +, fluorescence detected; -, fluorescence not detected; weak, weak signal, as determined from images of several experimental samples. (PDF) [file ppat.1010017.s002.pdf]

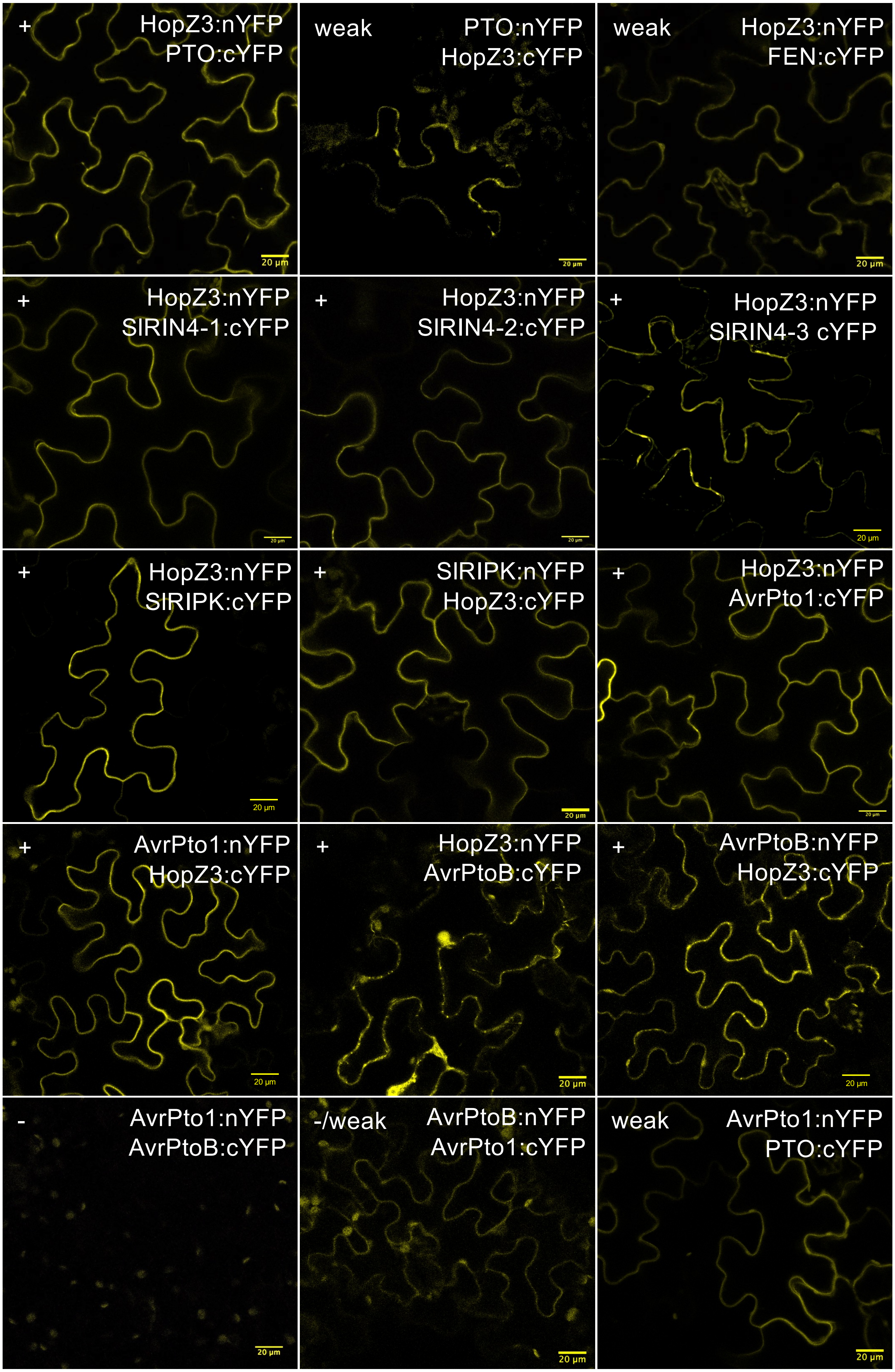
+
HopZ3:nYFP
PTO:cYFP
20 µm
weak
PTO:nYFP
HopZ3:cYFP
20 µm
weak
HopZ3:nYFP
FEN:cYFP
20 µm
+
HopZ3:nYFP
SlRIN4-1:cYFP
+
HopZ3:nYFP
SlRIN4-2:cYFP
+
HopZ3:nYFP
SlRIN4-3 cYFP
20 µm
+
HopZ3:nYFP
SlRIPK:cYFP
20 µm
+
SlRIPK:nYFP
HopZ3:cYFP
20 µm
+
HopZ3:nYFP
AvrPto1:cYFP
+
AvrPto1:nYFP
HopZ3:cYFP
20 µm
+
HopZ3:nYFP
AvrPtoB:cYFP
20 µm
+
AvrPtoB:nYFP
HopZ3:cYFP
20 µm
-
AvrPto1:nYFP
AvrPtoB:cYFP
20 µm
-/weak
AvrPtoB:nYFP
AvrPto1:cYFP
20 µm
weak
AvrPto1:nYFP
PTO:cYFP
20 µm

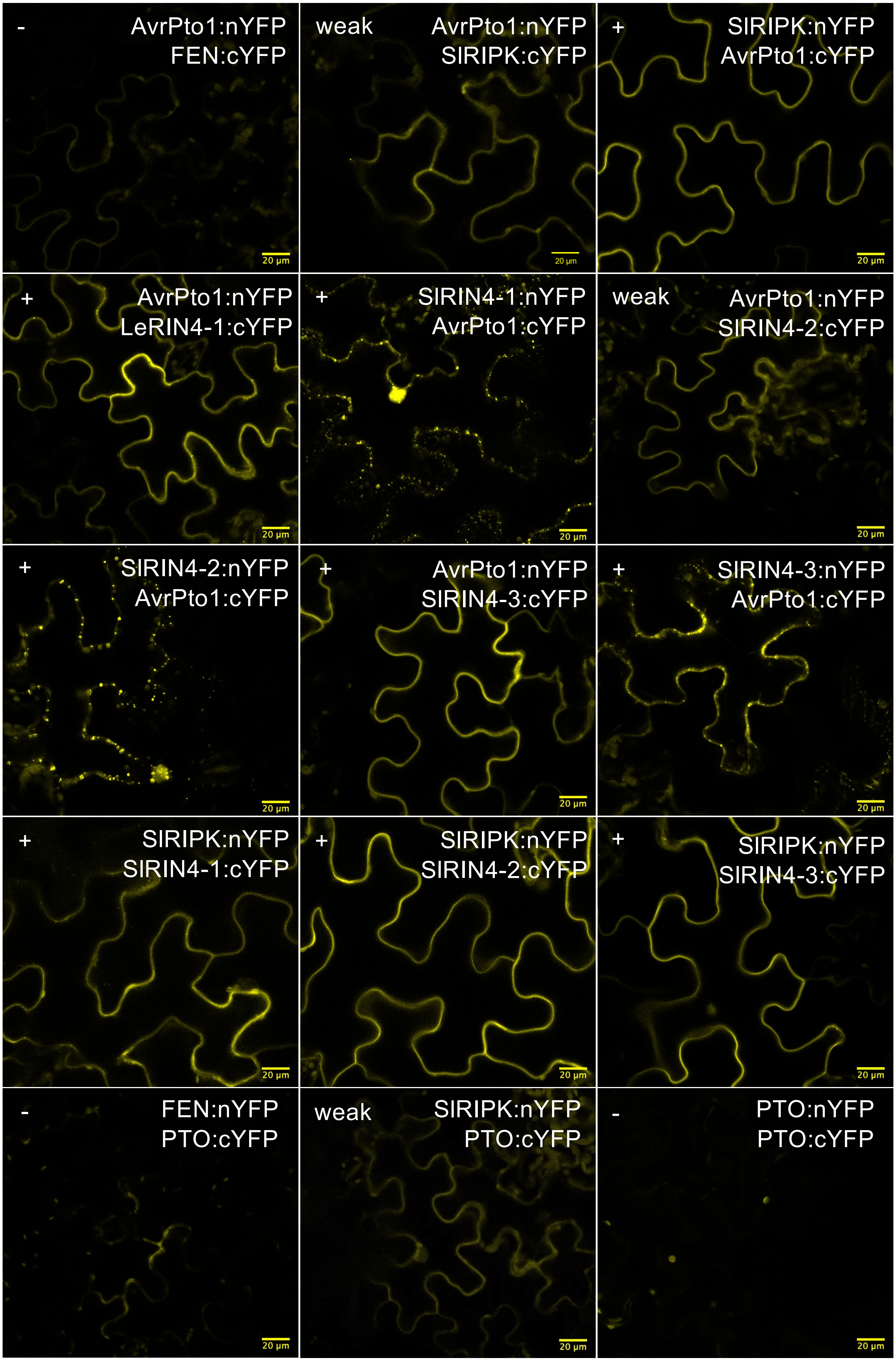
-
AvrPto1:nYFP
FEN:cYFP
weak
AvrPto1:nYFP
SlRIPK:cYFP
+
SlRIPK:nYFP
AvrPto1:cYFP
+
AvrPto1:nYFP
LeRIN4-1:cYFP
+
SlRIN4-1:nYFP
AvrPto1:cYFP
weak
AvrPto1:nYFP
SlRIN4-2:cYFP
+
SlRIN4-2:nYFP
AvrPto1:cYFP
+
AvrPto1:nYFP
SlRIN4-3:cYFP
+
SlRIN4-3:nYFP
AvrPto1:cYFP
+
SlRIPK:nYFP
SlRIN4-1:cYFP
+
SlRIPK:nYFP
SlRIN4-2:cYFP
+
SlRIPK:nYFP
SlRIN4-3:cYFP
-
FEN:nYFP
PTO:cYFP
weak
SlRIPK:nYFP
PTO:cYFP
-
PTO:nYFP
PTO:cYFP
20 μm

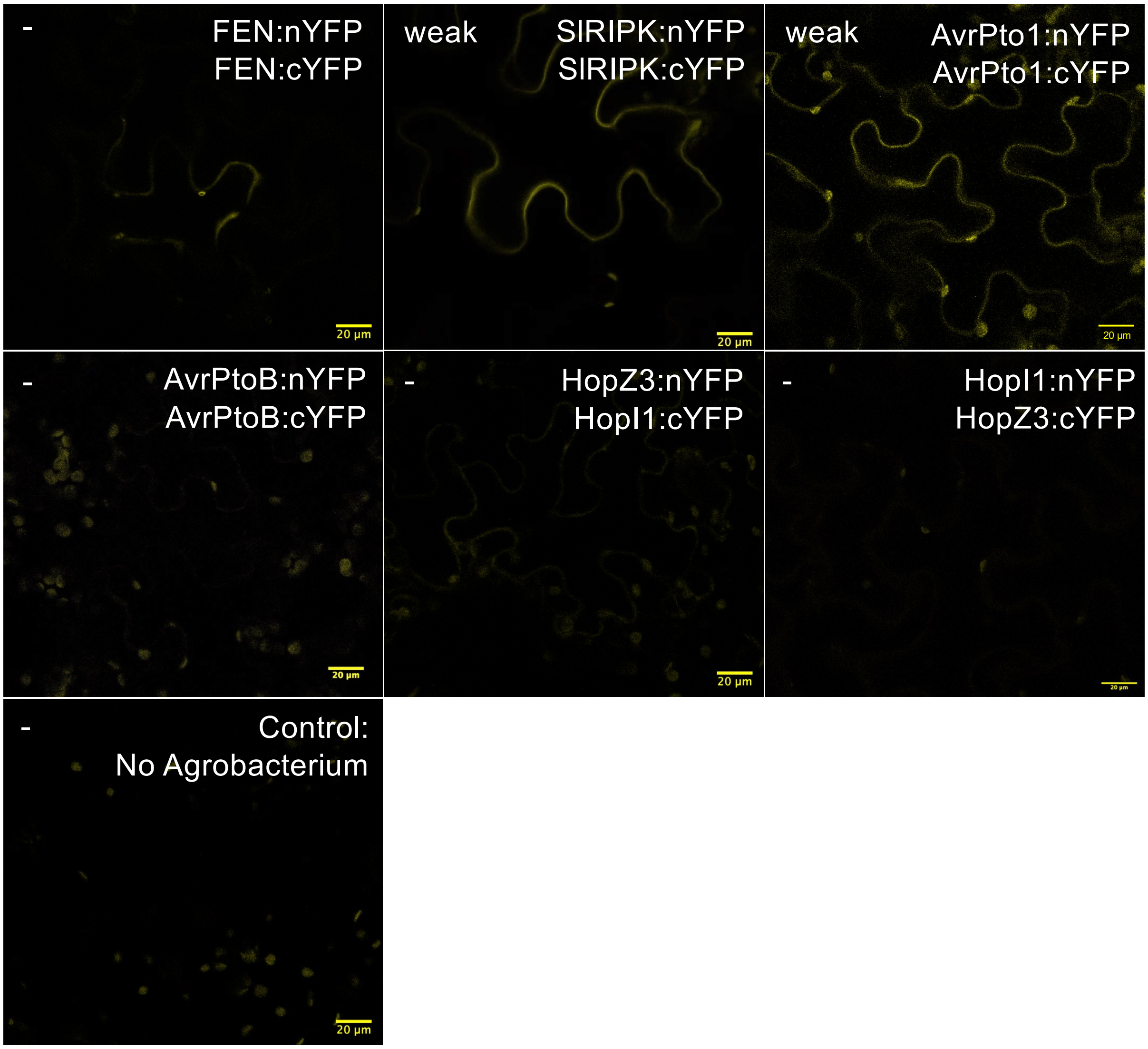

S2 Fig. Interaction between HopZ3, AvrPto1$_{Psy}$ and their potential interactors *in planta*. Interactions of HopZ3, AvrPto1$_{Psy}$, AvrPtoB$_{Psy}$, PTO, FEN, SlRIPK, SlRIN4-1, -2 and -3 were tested by BiFC. YFP fluorescence was imaged by confocal microscopy in epidermal *N. benthamina* cells co-infiltrated with mixtures of Agrobacteria harboring expression plasmids pG005 (protein:nYFP fusions) and pG006 (protein:cYFP fusions). Bar = 20 μm. Schematic overview of a subset of tested combinations is represented in Table 1. +, fluorescence detected; -, fluorescence not detected; weak, weak signal, as determined from images of several experimental samples.
