# Supplementary material for: Pseudomonas syringae effector HopZ3 suppresses the bacterial AvrPto1–tomato PTO immune complex via acetylation
Source: PLoS Pathog. 2021 Nov 1;17(11):e1010017. doi: 10.1371/journal.ppat.1010017 (PMC8584673; doi:10.1371/journal.ppat.1010017)
Supplement: S3 Fig — Empty Ni2+ resin or immobilized GST were used as negative controls for His- and GST- pull downs, respectively. Proteins were detected by Coomassie staining or immunoblotting. (A) Immobilized His-tagged AvrPto1Psy pulled down purified PTO-MBP. (B) Immobilized AvrPto1Psy -His was incubated with FEN-GST, showing no interaction. (C) Immobilized His-tagged AvrPto1Psy was incubated with SlRIN4-1-MBP showing weak interaction in one experiment and no interaction in two experiments. (D) Immobilized His-tagged AvrPto1Psy was incubated with SlRIN4-2-GST showing weak interaction in two of three experiments. (E) Immobilized His-tagged AvrPto1Psy was incubated with SlRIN4-3-GST. Interaction was not detected. (F) Immobilized GST-AvrPto1Psy or GST was mixed with His-tagged AvrPtoBPsy, showing that the two effectors interact. (PDF) [file ppat.1010017.s003.pdf]

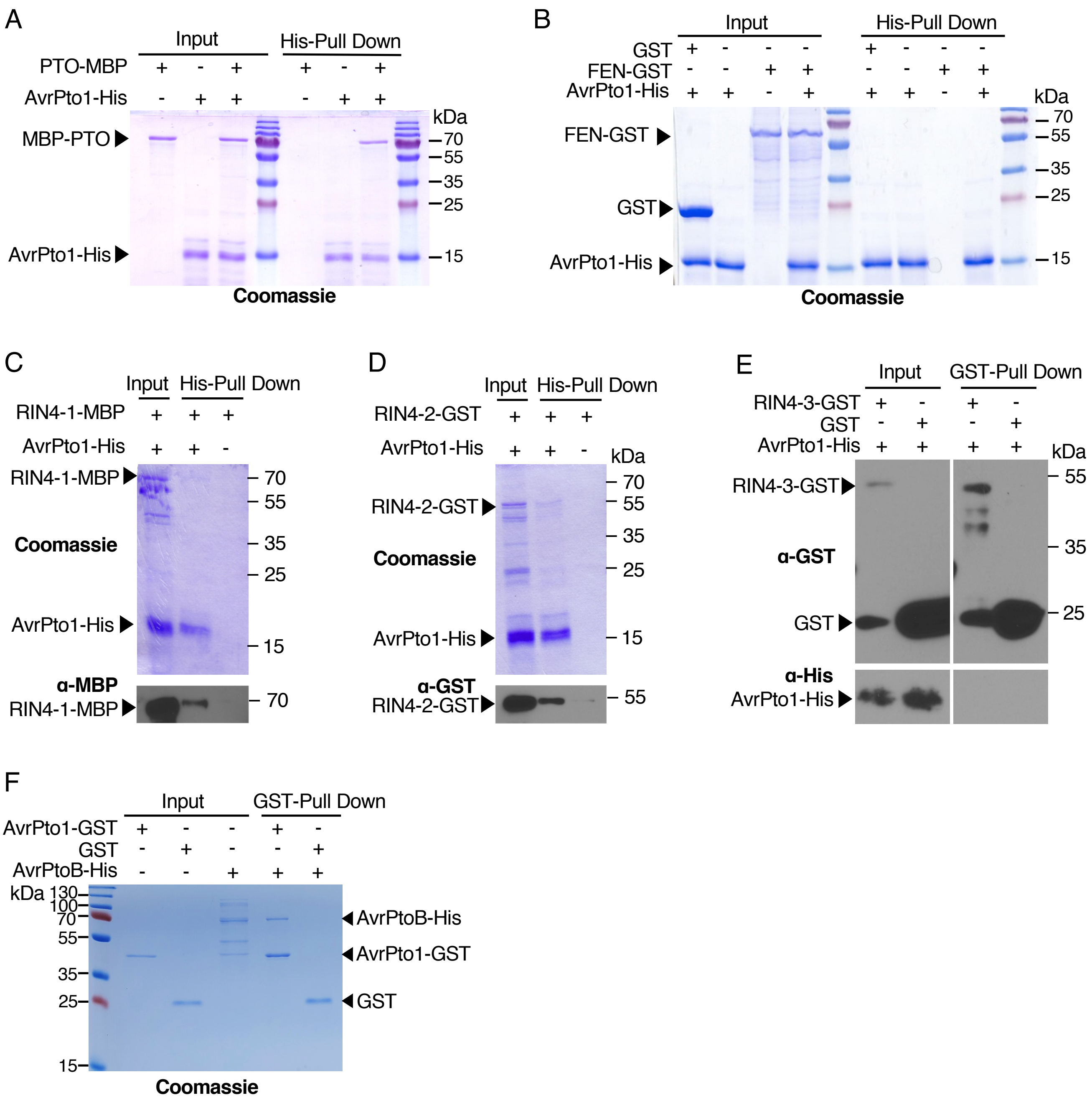

S3 Fig. Pull downs with recombinant tagged proteins to assess interaction between AvrPto1$_{Psy}$ and proteins in the PTO immune pathway. Empty Ni^{2+} resin or immobilized GST were used as negative controls for His- and GST pull downs, respectively. Proteins were detected by Coomassie staining or immunoblotting. (A) Immobilized His-tagged AvrPto1$_{Psy}$ pulled down purified PTO-MBP. (B) Immobilized AvrPto1$_{Psy}$-His was incubated with FEN-GST, showing no interaction. (C) Immobilized His-tagged AvrPto1$_{Psy}$ was incubated with SlRIN4-1-MBP showing weak interaction in 1 experiment and no interaction in 2 experiments. (D) Immobilized His-tagged AvrPto1$_{Psy}$ was incubated with SlRIN4-2-GST showing weak interaction in 2 of 3 experiments. (E) Immobilized His-tagged AvrPto1$_{Psy}$ was incubated with SlRIN4-3-GST. Interaction was not detected. (F) Immobilized GST-AvrPto1$_{Psy}$ or GST was mixed with His-tagged AvrPtoB$_{Psy}$, showing that the two effectors interact.
