# Supplementary material for: Pseudomonas syringae effector HopZ3 suppresses the bacterial AvrPto1–tomato PTO immune complex via acetylation
Source: PLoS Pathog. 2021 Nov 1;17(11):e1010017. doi: 10.1371/journal.ppat.1010017 (PMC8584673; doi:10.1371/journal.ppat.1010017)
Supplement: S4 Fig — MS/MS spectra show PTM of AvrPto1Psy expressed in N. benthamiana in the presence of HopZ3 or HopZ3_C300A. (A) Evidence of G2 myristoylation. (B) Acetylation of H125 and H130 in the presence of HopZ3. S136 was phosphorylated in both samples (HopZ3 and HopZ3_C300A). (C) Acetylation of T91 and S94 observed in the presence of HopZ3. (D–F) Acetylation of S147 and S149 in the presence of HopZ3 (D) reduced phosphorylation of these residues. Phosphorylation was observed in the presence of HopZ3_C300A (E–F). (PDF) [file ppat.1010017.s004.pdf]

AvrPto1

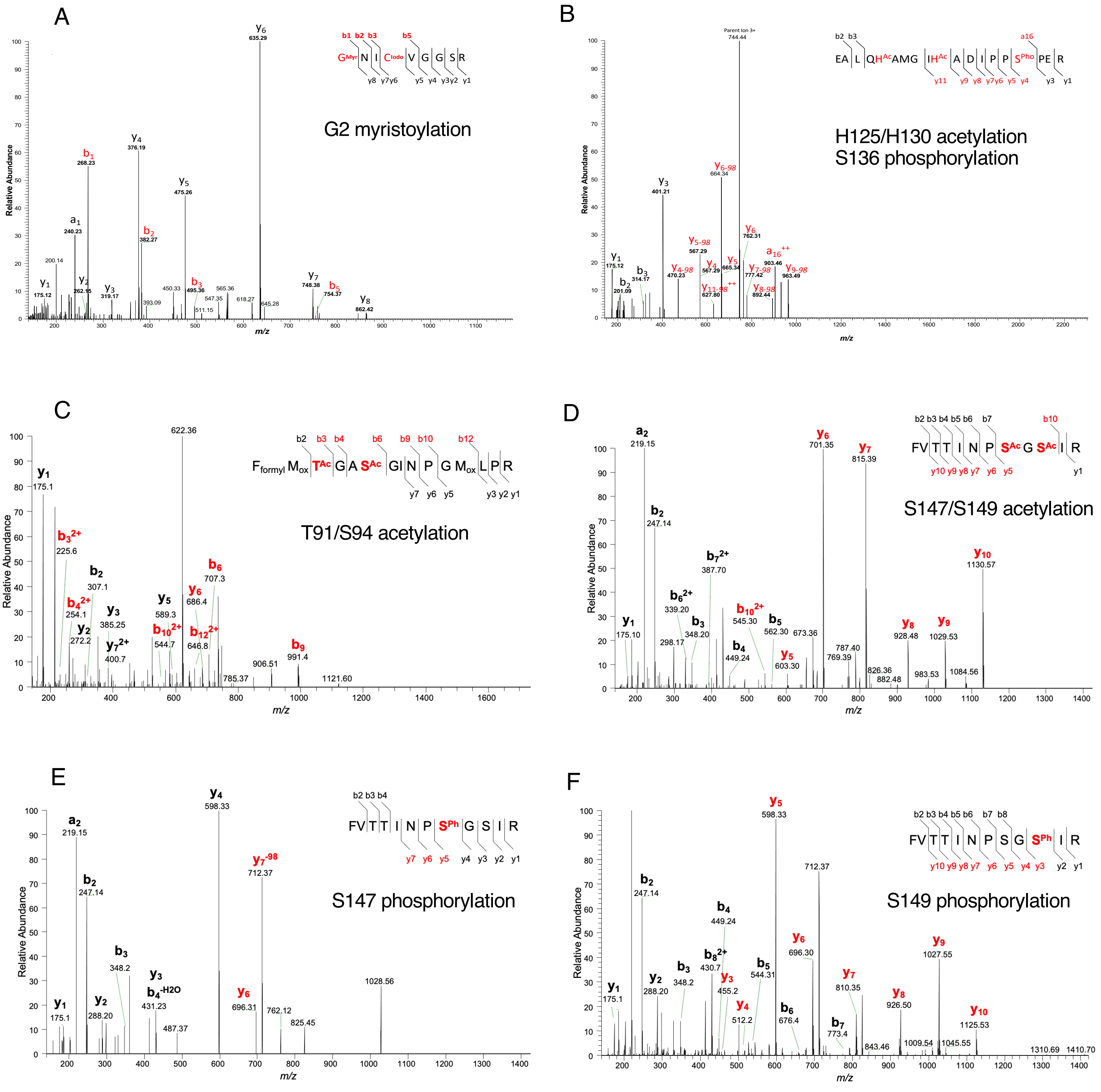

S4 Fig. HopZ3 acetylates multiple sites in AvrPto1$_{Psy}$ important for interaction with PTO and signaling. MS/MS spectra show PTM of AvrPto1$_{Psy}$ expressed in *N. benthamiana* in the presence of HopZ3 or HopZ3_C300A. (A) Evidence of G2 myristoylation. (B) Acetylation of H125 and H130 in the presence of HopZ3. S136 was phosphorylated in both samples (HopZ3 and HopZ3_C300A). (C) Acetylation of T91 and S94 observed in the presence of HopZ3. (D-F) Acetylation of S147 and S149 in the presence of HopZ3 (D) reduced phosphorylation of these residues. Phosphorylation was observed in the presence of HopZ3_C300A (E-F).
