# Supplementary material for: Pseudomonas syringae effector HopZ3 suppresses the bacterial AvrPto1–tomato PTO immune complex via acetylation
Source: PLoS Pathog. 2021 Nov 1;17(11):e1010017. doi: 10.1371/journal.ppat.1010017 (PMC8584673; doi:10.1371/journal.ppat.1010017)
Supplement: S5 Fig — PTMs were analyzed using mass spectrometry for PTO after in vitro acetylation reaction (A) or co-expressed with HopZ3 or HopZ3_C300A in N. benthamiana (B–E). (A–B) In both in vitro (A) and in planta (B) analyses T204, a key residue in the activation loop of the PTO kinase, was acetylated in the presence of HopZ3. (C) T199 acetylation in the presence of HopZ3. (D–E) Phosphorylation of S198 and T199 in the presence of HopZ3_C300A. (PDF) [file ppat.1010017.s005.pdf]

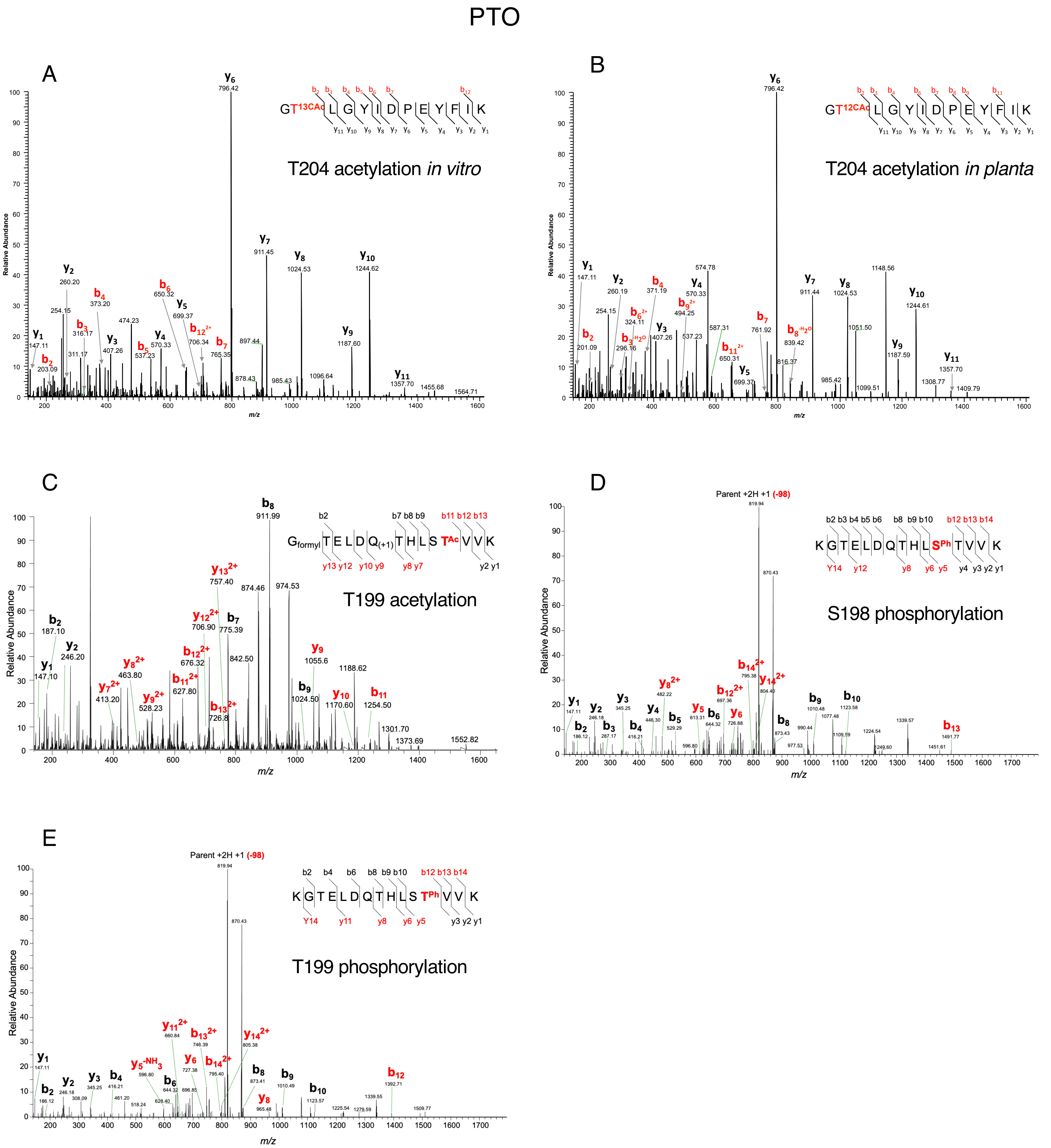

S5 Fig. HopZ3 acetylates key sites in the activation loop of PTO. PTMs were analyzed using mass spectrometry for PTO after *in vitro* acetylation reaction (A) or co-expressed with HopZ3 or HopZ3_C300A *in N. benthamiana* (B-E). (A-B) In both *in vitro* (A) and *in planta* (B) analyses T204, a key residue in the activation loop of the PTO kinase, was acetylated in the presence of HopZ3. (C) T199 acetylation in the presence of HopZ3. (D-E) Phosphorylation of S198 and T199 in the presence of HopZ3_C300A.
