# Supplementary material for: Pseudomonas syringae effector HopZ3 suppresses the bacterial AvrPto1–tomato PTO immune complex via acetylation
Source: PLoS Pathog. 2021 Nov 1;17(11):e1010017. doi: 10.1371/journal.ppat.1010017 (PMC8584673; doi:10.1371/journal.ppat.1010017)
Supplement: S6 Fig — AvrPto1Psy-GFP variants were transiently expressed in N. benthamiana infiltrated with Agrobacterium at OD = 0.2 or 0.4 and sprayed with dexamethasone. (A) Only AvrPto1Psy_T91A induced delayed cell death compared to wild-type AvrPto1Psy. All variants were expressed to similar levels. A number of infiltrated areas with cell death per total number of samples is shown in the tables. (B) H125, H130 and double mutant of AvrPto1Psy induced cell death in N. benthamiana. (PDF) [file ppat.1010017.s006.pdf]

A

OD=0.2 OD=0.4

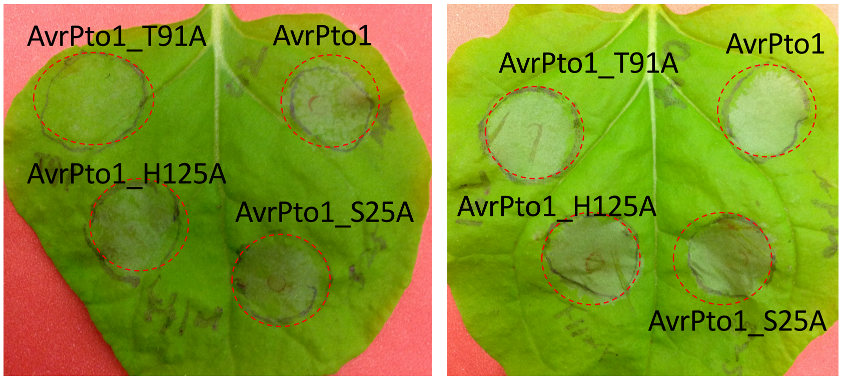

16 h after 30 μM Dex treatment

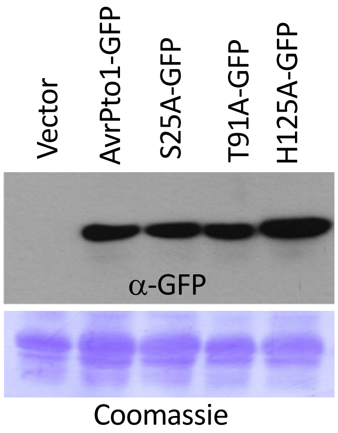

	O.D=0.2	O.D=0.4
AvrPto1	4/4	4/4
AvrPto1_S25A	4/4	4/4
AvrPto1_T91A	1/4	4/4
AvrPto1_H125A	4/4	4/4

B

OD=0.2

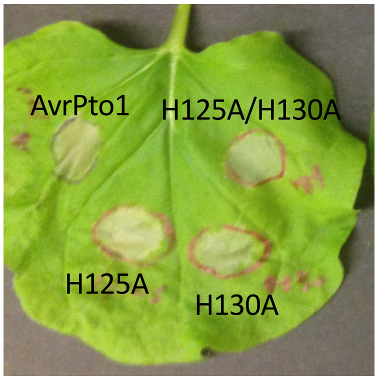

	O.D=0.2
AvrPto1	6/6
AvrPto1_H125A	6/6
AvrPto1_H130A	6/6
AvrPto1_H125A/H130A	6/6

S6 Fig. Effect of mutations of AvrPto1$_{Psy}$ acetylation sites on cell death induction in *N. benthamiana*. AvrPto1$_{Psy}$-GFP variants were transiently expressed in *N. benthamiana* infiltrated with Agrobacterium at OD=0.2 or 0.4 and sprayed with dexamethasone. (A) Only AvrPto1$_{Psy}$_T91A induced delayed cell death compared to wild-type AvrPto1$_{Psy}$. All variants were expressed to similar levels. A number of infiltrated areas with cell death per total number of samples is shown in tables. (B) H125, H130 and double mutant of AvrPto1$_{Psy}$ induced cell death in *N. benthamiana*.
