# Supplementary material for: Pseudomonas syringae effector HopZ3 suppresses the bacterial AvrPto1–tomato PTO immune complex via acetylation
Source: PLoS Pathog. 2021 Nov 1;17(11):e1010017. doi: 10.1371/journal.ppat.1010017 (PMC8584673; doi:10.1371/journal.ppat.1010017)
Supplement: S8 Fig — Modifications in SlRIPK were determined in vitro, modifications in AtRIPK are from [9] (in vitro and in planta). Residues acetylated by HopZ3 are bold and highlighted in yellow; phosphorylation sites are underlined; known sites in AtRIPK important for activity (K122; S251/T252 which correspond to S198/T199 in PTO) [9] are circled in red; sites corresponding to T204 in PTO are circled in blue. * (asterisk)—fully conserved residues,: (colon)—conservation between groups of strongly similar properties,. (period)—conservation between groups of weakly similar properties. (PDF) [file ppat.1010017.s008.pdf]

```
SlRIPK  MATCG-IDWKSVLPNCFKGNNVRSEAKVMENSK----QMNSDHHRLAFSDISTDSRSVLI 55
AtRIPK  MAVKKKVSWRSLIVGCLGDPETLMASSKKPKRKNDVIKKQSSFQRLSILDMSNPSSNTLS 60
        **.   :.*:*:: .*: . :.   :.   : *    : :*..:**:: *:*. * ..*
SlRIPK  SLDDLSSNAVIGSNLHVFTYEELKLITSDFSSANFLGKGGFGPVHKGFIDDKIKPGLDAQ 115
AtRIPK  --EDLS-ISLAGSDLHVFTLAELKVITQSFSSTNFLGEGGFGPVHKGFIDDKLRPGLKAQ 117
          :***  :: **:*****  ***:**..***:****:**************::***.**
SlRIPK  PVAVKLLDLDGNQGHQEWLTEVVFLGQLRHHHLVKLIGYCWEEEQRLLVYEYMARGNLED 175
AtRIPK  PVAVKLLDLEGLQGHREWLTEVMFLGQLKHKNLVKLIGYCCEEEHRTLVYEFMPRGSLEN 177
        *********:* ***:******:*****:*::******** ***:* ****:*.**.**:
SlRIPK  QLFSRYSSCLPWLTRIKIMVGAAKGLAFLHGEEKPVIYRDFKASNILLDSDYRAKLSDFG 235
AtRIPK  QLFRRYSASLPWSTRMKIAHGAATGLQFLHEAENPVIYRDFKASNILLDSDYTAKLSDFG 237
        *** ***:.*** **:**  ***.** ***  *:****************** *******
SlRIPK  LAKDGPEGDDTHVSTRVMGTHGYAAPEYIMTGHLTSKSDVYSFGVVLLELITGRRAMDKK 295
AtRIPK  LAKDGPEGDDTHVSTRVMGTQGYAAPEYIMTGHLTARSDVYSFGVVLLELLTGRRSVDKK 297
        ********************:**************::*************:****::***
SlRIPK  RPLKERILVDWARPMLRDPHKLDRIMDPRLEGQYSTQGAKKVAALAYQCLSHHPRSRPTM 355
AtRIPK  RSSREQNLVDWARPMLNDPRKLSRIMDPRLEGQYSETGARKAATLAYQCLSHRPKNRPCM 357
        *. :*: *********.**:**.************  **:*.*:********:*:.** *
SlRIPK  SNIVKILEPVLDMKDIPMGPFVYVVPSSKPDKGTEIGELKTKVNDENKAGVRENEVDNAG 415
AtRIPK  SAVVSILNDLKDYNDIPMGTFTYTVPN-TPDNKEDDGRVGNKPRKSSHHHHHQQQQSNHP 416
        * :*.**: : * :*****.*.*.**. .**:  : *.: .* ....:   :::: .*
SlRIPK  ENREDGNAKQRRVGHRYKHRLKTDASVYSDTHLYHKTVKHERTNKLNSY 464
AtRIPK  RSSPSPTTKSPSP-TAKSPRNSTENHRRTLRNGVNSPLRSEAGGERY-- 462
        ..  . .:*.       . * .*:    :  :  :..:: *  .:
```

S8 Fig. HopZ3 acetylates SlRIPK residues important for activity. Modifications in SlRIPK were determined *in vitro*, modifications in AtRIPK are from [1] (*in vitro* and *in planta*). Residues acetylated by HopZ3 are bold and highlighted in yellow; phosphorylation sites are underlined; known sites in AtRIPK important for activity (K122; S251/T252 which correspond to S198/T199 in PTO) [1] are circled in red; sites corresponding to T204 in PTO are circled in blue. * (asterisk) - fully conserved residues, : (colon) - conservation between groups of strongly similar properties, . (period) - conservation between groups of weakly similar properties.

References

1. Lee J, Manning AJ, Wolfgeher D, Jelenska J, Cavanaugh KA, Xu H, et al. Acetylation of an NB-LRR plant immune-effector complex suppresses immunity. Cell Rep. 2015;13(8):1670-82. doi: 10.1016/j.celrep.2015.10.029. PubMed PMID: 26586425.
