# Supplementary material for: Pseudomonas syringae effector HopZ3 suppresses the bacterial AvrPto1–tomato PTO immune complex via acetylation
Source: PLoS Pathog. 2021 Nov 1;17(11):e1010017. doi: 10.1371/journal.ppat.1010017 (PMC8584673; doi:10.1371/journal.ppat.1010017)
Supplement: S9 Fig — (A) PTO and FEN phosphorylate tomato SlRIN4-1. Kinase assays showing an increasing amount of PTO and FEN autophosphorylation and transphosphorylation of SlRIN4-1. (B-C) SlRIPK and FEN phosphorylate SlRIN4-3. The time course of the in vitro kinase reactions is shown. Purified SlRIPK (B) or FEN (C) were incubated in kinase buffer with or without SlRIN4-3 as a substrate. At indicated time points, aliquots of the reaction were taken out and separated by SDS-PAGE. (PDF) [file ppat.1010017.s009.pdf]

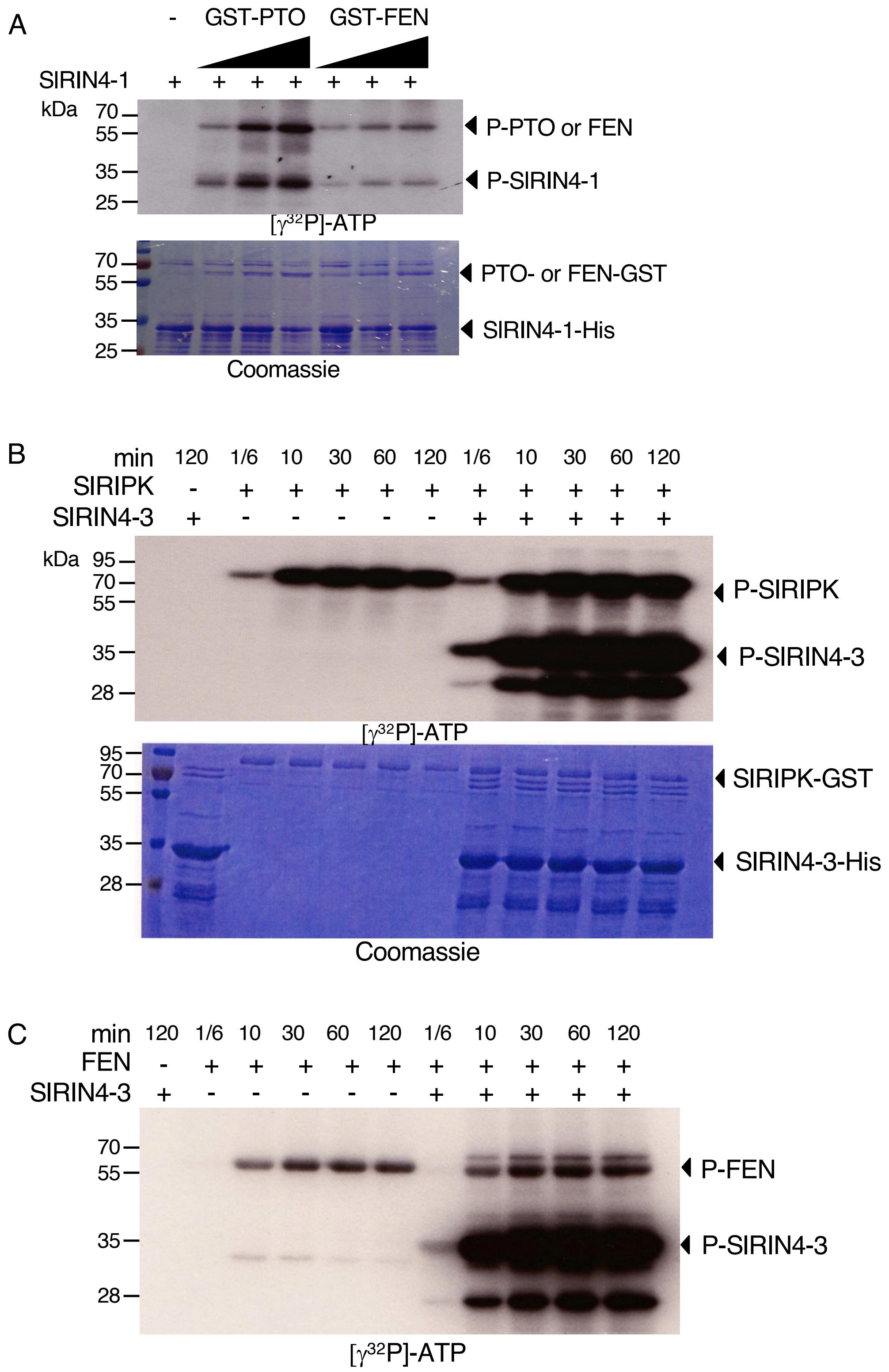

S9 Fig. Kinases in the PTO family can phosphorylate tomato RIN4s. (A) PTO and FEN phosphorylate tomato SlRIN4-1. Kinase assays showing an increasing amount of PTO and FEN autophosphorylation and transphosphorylation of SlRIN4-1. (B-C) SlRIPK and FEN phosphorylate SlRIN4-3. The time course of the *in vitro* kinase reactions is shown. Purified SlRIPK (B) or FEN (C) were incubated in kinase buffer with or without SlRIN4-3 as a substrate. At indicated time points, aliquots of the reaction were taken out and separated by SDS-PAGE.
