# Supplementary material for: Pseudomonas syringae effector HopZ3 suppresses the bacterial AvrPto1–tomato PTO immune complex via acetylation
Source: PLoS Pathog. 2021 Nov 1;17(11):e1010017. doi: 10.1371/journal.ppat.1010017 (PMC8584673; doi:10.1371/journal.ppat.1010017)
Supplement: S1 Table — PTMs were determined either in vitro, using purified recombinant AvrPto1Psy after 13C-acetylation by HopZ3/HopZ3_C300A, or in planta, by co-expressing AvrPto1Psy and HopZ3/HopZ3_C300A in N. benthamiana, followed by immunoprecipitation. Numbers indicate enrichment (fold change) of acetylation in the presence of HopZ3 vs. HopZ3_C300A. Red shading: significant (>50%) increase of acetylation with HopZ3. Blue shading: significant decrease of phosphorylation in planta in the presence of HopZ3. Residues known to be important for AvrPto signaling or interaction with PTO are in bold. + indicates phosphorylation found in a recombinant protein (in vitro) or in planta. Z3: acetylation found only in AvrPto1Psy treated or co-expressed with HopZ3 and not HopZ3_C300A. Ac: acetylation; Phos: phosphorylation; Myr: myristoylation; exp: experiment. *Some spectra do not distinguish these 2 close residues. #In planta sites with acetylation above 25% in the presence of HopZ3. (PDF) [file ppat.1010017.s010.pdf]

S1 Table. AvrPto1$_{Psy}$ PTMs *in vitro and in planta*.

	In vitro		*In planta*				
Site	Ac Z3/CA	Phos	Myr	Ac Z3/CA		Phos	
			exp 1,2	exp 1	exp 2	exp 1	exp 2
G2			+				
S9	2.7	+					
H13	2.2				Z3		
S17	1.5	+			Z3	+	+
S22	5.9					+	+
S25	1.9	+		2	Z3	+	+
T32						+	+
S33						+	+
**S46**		+		15	5		
S58		+		Z3	Z3		
S59		+		3	14		
S67*	3.8				Z3		
S68*	1.2				0.8	+	+
T79	1.9			Z3	Z3		
H87	0.9			Z3			
**T91**	2	+		Z3#	18#		
**S94**	1.6			Z3#	20#		
S111	2.2				Z3		
S114	2.4				Z3		
S117	1.8	+			Z3		
H125	2.2			2#	Z3#		
H130	2.2			2	Z3		
S136						+	+
T142*	Z3	+			Z3	+	
T143*		+				+	
**S147**	1.6	+		1.6	Z3	+	+
**S149**	11	+		2	Z3	+	+
T154	19	+					
S158	1.4					+	+
H160	1.4						

PTMs were determined either *in vitro*, using purified recombinant AvrPto1$_{Psy}$ after ^{13}C-acetylation by HopZ3/HopZ3_C300A, or *in planta*, by co-expressing AvrPto1$_{Psy}$ and HopZ3/HopZ3_C300A in *N. benthamiana*, followed by immunoprecipitation. Numbers indicate enrichment (fold change) of acetylation in the presence of HopZ3 vs. HopZ3_C300A. Red shading: significant (>50%) increase of acetylation with HopZ3. Blue shading: significant decrease of phosphorylation *in planta* in the presence of HopZ3. Residues known to be important for AvrPto signaling or interaction with PTO are in **bold**. + indicates phosphorylation found in a recombinant protein (*in vitro*) or *in planta*. Z3: acetylation found only in AvrPto1$_{Psy}$ treated or co-expressed with HopZ3 and not HopZ33_C300A. Ac: acetylation; Phos: phosphorylation; Myr: myristoylation; exp: experiment. *Some spectra do not distinguish these 2 close residues. #*In planta* sites with acetylation above 25% in the presence of HopZ3.
