# Supplementary material for: Pseudomonas syringae effector HopZ3 suppresses the bacterial AvrPto1–tomato PTO immune complex via acetylation
Source: PLoS Pathog. 2021 Nov 1;17(11):e1010017. doi: 10.1371/journal.ppat.1010017 (PMC8584673; doi:10.1371/journal.ppat.1010017)
Supplement: S2 Table — PTMs were determined either in vitro, using purified recombinant PTO after 13C-acetylation by HopZ3/HopZ3_C300A, or in planta, by co-expressing PTO and HopZ3/HopZ3_C300A in N. benthamiana, followed by immunoprecipitation. Numbers indicate enrichment (fold change) of acetylation in the presence of HopZ3 vs. HopZ3_C300A. Red shading: significant (>50%) increase of acetylation with HopZ3. Blue shading: significant decrease of phosphorylation in planta in the presence of HopZ3. Residues important for PTO signaling or interaction with AvrPto are in bold. + indicates phosphorylation found in a recombinant protein (in vitro) or in planta. Z3: acetylation found only in PTO treated or co-expressed with HopZ3 and not HopZ33_C300A.; Ac: acetylation; Phos: phosphorylation; exp: experiment. *Some spectra do not distinguish these 2 close residues. #In planta sites with acetylation above 25% in the presence of HopZ3. (PDF) [file ppat.1010017.s011.pdf]

S2 Table. PTO PTMs *in vitro* and *in planta*.

	In vitro		*In planta*			
Site	Ac Z3/CA	Phos	Ac Z3/CA		Phos	
			exp 1	exp 2	exp 1	exp 2
T9*			Z3	1.8		
S11*			Z3	2		
**T38**		+			+	
K123			Z3	Z3#		
T133		+			+	+
S135					+	+
S137					+	+
T181					+	
T190					+	+
T195		+			+	
**S198**		+			+	+
**T199**		+	Z3	Z3#	+	
K202			Z3	1		
**T204**	Z3		Z3#	Z3#		

PTMs were determined either *in vitro*, using purified recombinant PTO after ^{13}C-acetylation by HopZ3/HopZ3_C300A, or *in planta*, by co-expressing PTO and HopZ3/HopZ3_C300A in *N. benthamiana*, followed by immunoprecipitation. Numbers indicate enrichment (fold change) of acetylation in the presence of HopZ3 vs. HopZ3_C300A. Red shading: significant (>50%) increase of acetylation with HopZ3. Blue shading: significant decrease of phosphorylation *in planta* in the presence of HopZ3. Residues important for PTO signaling or interaction with AvrPto are in **bold**. + indicates phosphorylation found in a recombinant protein (*in vitro*) or *in planta*. Z3: acetylation found only in PTO treated or co-expressed with HopZ3 and not HopZ33_C300A.; Ac: acetylation; Phos: phosphorylation; exp: experiment. *Some spectra do not distinguish these 2 close residues. # *In planta* sites with acetylation above 25% in the presence of HopZ3.
