# Supplementary material for: Pseudomonas syringae effector HopZ3 suppresses the bacterial AvrPto1–tomato PTO immune complex via acetylation
Source: PLoS Pathog. 2021 Nov 1;17(11):e1010017. doi: 10.1371/journal.ppat.1010017 (PMC8584673; doi:10.1371/journal.ppat.1010017)
Supplement: S3 Table — PTMs were determined using purified recombinant SlRIN4s after in vitro 13C-acetylation by HopZ3/HopZ3_C300A. Numbers indicate enrichment (fold change) of 13C-acetylation in the presence of HopZ3 vs. HopZ3_C300A. Z3: acetylation found only in SlRIN4 treated with HopZ3. Red shading: significant (>50%) increase of modification with HopZ3. + indicates phosphorylation found in a recombinant protein. Ac: acetylation; Phos: phosphorylation. (PDF) [file ppat.1010017.s012.pdf]

S3 Table. SlRIN4s PTMs *in vitro*.

Protein	Site	Ac Z3/CA	Phos
SlRIN4_1	S88	Z3	
	T128	Z3	
	S153	Z3	
	K167	1.4	
	S188		+
	S207	1.4	
	K248	Z3	
SlRIN4_2	T18		+
	K34	2	
	S144		+
	S180		+
	T185		+
	K210	3	
SlRIN4_3	K48	Z3	
	K114	Z3	
	S213		+
	T239		+
	T276		+
	S284		+

PTMs were determined using purified recombinant SlRIN4s after *in vitro* ^{13}C-acetylation by HopZ3/HopZ3_C300A. Numbers indicate enrichment (fold change) of ^{13}C-acetylation in the presence of HopZ3 vs. HopZ3_C300A. Z3: acetylation found only in SlRIN4 treated with HopZ3. Red shading: significant (>50%) increase of modification with HopZ3. + indicates phosphorylation found in a recombinant protein. Ac: acetylation; Phos: phosphorylation.
