# Supplementary material for: Pseudomonas syringae effector HopZ3 suppresses the bacterial AvrPto1–tomato PTO immune complex via acetylation
Source: PLoS Pathog. 2021 Nov 1;17(11):e1010017. doi: 10.1371/journal.ppat.1010017 (PMC8584673; doi:10.1371/journal.ppat.1010017)
Supplement: S4 Table — PTMs were determined using purified recombinant SlRIPK after in vitro 13C-acetylation by HopZ3/HopZ3_C300A. Numbers indicate enrichment (fold change) of 13C-acetylation in the presence of HopZ3 vs. HopZ3_C300A. Z3: acetylation found only in SlRIPK treated with HopZ3. Red shading: significant (>50%) increase of modification with HopZ3. + indicates phosphorylation found in a recombinant protein. Ac: acetylation; Phos: phosphorylation. (PDF) [file ppat.1010017.s013.pdf]

S4 Table. SlRIPK PTMs *in vitro*.

Site	Ac Z3/CA	Phos
S10	1.4	+
K17	Z3	
S31		+
H38/H39	Z3	
S44		+
S47		+
S52		+
S56		+
T82	2	+
S83	1.6	+
S86/S87		+
K120	Z3	
H130	2	
T135	2	+
S182	1.4	+
S219	1.5	+
S232		+
T246	1.8	+
H247	2.2	
S249	Z3	+
T250	Z3	+
T255	1.6	+
H256	1.8	
T266		+
S330		+
T354		+
S382		+
T389		+
T396		+
S444		+
T446		+
T452		+

PTMs were determined using purified recombinant SlRIPK after *in vitro* ^{13}C-acetylation by HopZ3/HopZ3_C300A. Numbers indicate enrichment (fold change) of ^{13}C-acetylation in the presence of HopZ3 vs. HopZ3_C300A. Z3: acetylation found only in SlRIPK treated with HopZ3. Red shading: significant (>50%) increase of modification with HopZ3. + indicates phosphorylation found in a recombinant protein. Ac: acetylation; Phos: phosphorylation.
