# Supplementary material for: Pseudomonas syringae effector HopZ3 suppresses the bacterial AvrPto1–tomato PTO immune complex via acetylation
Source: PLoS Pathog. 2021 Nov 1;17(11):e1010017. doi: 10.1371/journal.ppat.1010017 (PMC8584673; doi:10.1371/journal.ppat.1010017)
Supplement: S5 Table — (PDF) [file ppat.1010017.s014.pdf]

S5 Table. Bacterial and yeast strains.

Strain	Description	Antibiotic[a]	Reference/ Source
Escherichia coli			
DH5α	F⁻ Φ80d*lacZ*Δ*M15* Δ(*lac*ZYA-*argF*)*U169 endA1 recA1 hsdR17* ($r_K^-m_K^+$) *deoR thi*-1 *supE44* λ⁻ *gyrA96 relA1*	Nal^R	Invitrogen
BL21 (DE3)	F⁻ *ompT gal dcm lon hsdS*$_B$(r_B^- m_B^-) λ(DE3 [*lacI lacUV5*-T7 gene 1 *ind1 sam7 nin5*])		Stratagene
Saccharomyces cerevisiae			
AH109	MATa, trp1-901, leu2-3, 112, ura3-52, his3-200, gal4Δ, gal80Δ, LYS2::GAL1$_{UAS}$-GAL1$_{TATA}$-HIS3, GAL2$_{UAS}$-GAL2$_{TATA}$-ADE2, URA3::MEL1$_{UAS}$-MEL1$_{TATA}$-lacZ		Clontech
Y187	*MATα, ura3-52, his3-200, ade2-101, trp1-901, leu2-3, 112, gal4Δ, met-, gal80Δ, MEL1, URA3::GAL1*$_{USA}$*-GAL1*$_{TATA}$*-lacZ*		Clontech
Pseudomonas syringae			
*Psy*B728a	*Pseudomonas syringae* pv. *syringae* B728a	Rif^R	[1]
*Psy*B728a *ΔhopZ3*	HopZ3 knock-out mutant of *Pseudomonas syringae* pv. *syringae* B728a	Rif^R	[1]
*Psy*B728a *ΔAvrPto1*	AvrB3 knock-out mutant of *Pseudomonas syringae* pv. *syringae* B728a	Rif^R	This study
*Psy*B728a *ΔHopZ3ΔAvrPto1*	HopZ3/AvrPto1 double knock-out mutant of *Pseudomonas syringae* pv. *syringae* B728a	Rif^R	This study
Agrobacteriun tumefaciens			
GV3101 (pCH32)	Used for *Agrobacterium*-mediated transient expression in *N. benthamiana*	Gm^R/Rif^R	
C58C1 (pMP90)	Used for *Agrobacterium*-mediated transient expression in *N. benthamiana*	Tet^R	

[a] Nal^R, nalidixic acid resistance; Rif^R, rifampicin resistance; Gm^R, gentamycin resistance; Tet^R, tetracycline resistance.

References

1. Vinatzer BA, Teitzel GM, Lee MW, Jelenska J, Hotton S, Fairfax K, et al. The type III effector repertoire of Pseudomonas syringae pv. syringae B728a and its role in survival and disease on host and non-host plants. Mol Microbiol. 2006;62(1):26-44. doi: 10.1111/j.1365-2958.2006.05350.x. PubMed PMID: 16942603.
