# Supplementary material for: Pseudomonas syringae effector HopZ3 suppresses the bacterial AvrPto1–tomato PTO immune complex via acetylation
Source: PLoS Pathog. 2021 Nov 1;17(11):e1010017. doi: 10.1371/journal.ppat.1010017 (PMC8584673; doi:10.1371/journal.ppat.1010017)
Supplement: S6 Table — (PDF) [file ppat.1010017.s015.pdf]

S6 Table. Plasmid vectors.

Plasmid	Parent vector	Description	Antibiotic[a]	Reference
pBAV154	pTA7001	Gateway-compatible plant expression binary vector (*dex* inducible promoter and C-terminal HA-tag)	Km^R / Cm^R /$BASTA^R$	[1]
pBAV150	pTA7001	Gateway-compatible plant expression binary vector (*dex* inducible promoter and C-terminal GFP-tag)	Km^R / Cm^R /$BASTA^R$	[1]
pTA7001		Vector control for *Agrobacterium*		[1]
pBAV226	pME6010	Gateway-compatible *P. syringae* expression vector (*nptII* promoter, low copy plasmid, C-terminal HA-Tag)	Tet^R/ Cm^R	[1]
pME6010		Vector control for *P. syringae*		[1]
pMTN1907		Gateway™ vector for making unmarked deletions in *P. syringae*. SacB cassette	Tet^R	[2]
pDONR207		Gateway™ cloning vector	Gm^R/ Cm^R	Invitrogen
pET DUET		T7 promoter, N-terminal His-tag or C-terminal S-tag	Amp^R	Novagen
pET28a		T7 promoter, N-terminal His-tag and T7-tag, optional C-terminal His-tag	Km^R	Novagen
pGEX 4T-1		tac promoter and N-terminal GST-tag with thrombin cleavage site	Amp^R	Amersham/ Pharmacia
pGEX 4T-3 with rTEV	pGEX 4T-3	tac promoter and N-terminal GST-tag, pGEX 4T-3 with rTEV site	Amp^R	from K. Orth (UT Southwestern)
pMAL-c5X		tac promoter and N-terminal MBP fusion	Amp^R	NE BioLabs
pG005	pGreen	BIFC gateway binary vector 35S promoter, GW, YFP N-terminus	Sp^R/ Cm^R/Hyg^R	NASC
pG006	pGreen	BIFC gateway binary vector, 35S promoter, GW, YFP C-terminus	Sp^R/ Cm^R /Hyg^R	NASC
pLaw10	pGBK T7	Yeast two-hybrid GAL4 BD vector, ADH1 promoter, N-terminal c-Myc tag. Gateway compatible derivative of pGBKT7 (Clontech).	Km^R/ Cm^R TRP	[3]
pLaw11	pGAD7	Yeast two-hybrid GAL4 AD vector, ADH1 promoter, N-terminal HA tag. Gateway compatible derivative of pGADT7 (Clontech).	Amp^R/ Cm^R /LEU	[3]

[a] Amp^R, ampicillin resistance; $BASTA^R$, BASTA (glufosinate ammonium) resistance; Cm^R, chloroamphenicol resistance; Gm^R, gentamycin resistance; Hyg^R, Hygromycin resistance; Km^R, kanamycin resistance; Tet^R, tetracycline resistance; Sp^R, spectinomycin resistance.

References

1. Vinatzer BA, Teitzel GM, Lee MW, Jelenska J, Hotton S, Fairfax K, et al. The type III effector repertoire of Pseudomonas syringae pv. syringae B728a and its role in survival and disease on host and non-host plants. Mol Microbiol. 2006;62(1):26-44. doi: 10.1111/j.1365-2958.2006.05350.x. PubMed PMID: 16942603.

2. Baltrus DA, Nishimura MT, Dougherty KM, Biswas S, Mukhtar MS, Vicente J, et al. The molecular basis of host specialization in bean pathovars of Pseudomonas syringae. Mol Plant Microbe Interact. 2012;25(7):877-88. doi: 10.1094/MPMI-08-11-0218. PubMed PMID: 22414441.

3. Lee J, Manning AJ, Wolfgeher D, Jelenska J, Cavanaugh KA, Xu H, et al. Acetylation of an NB-LRR plant immune-effector complex suppresses immunity. Cell Rep. 2015;13(8):1670-82. doi: 10.1016/j.celrep.2015.10.029. PubMed PMID: 26586425.
