# Supplementary material for: Pseudomonas syringae effector HopZ3 suppresses the bacterial AvrPto1–tomato PTO immune complex via acetylation
Source: PLoS Pathog. 2021 Nov 1;17(11):e1010017. doi: 10.1371/journal.ppat.1010017 (PMC8584673; doi:10.1371/journal.ppat.1010017)
Supplement: S7 Table — Mutated codons are underlined. (PDF) [file ppat.1010017.s016.pdf]

S7 Table. PCR primer sequences used in this study.

Primer name	Primer sequence (5'→3')
AvrPto1$_{Psy}$ 5'F1	AAAAAGCAGGCTTCAACTGGTTGCGCAAGACTGA
AvrPto1$_{Psy}$ 5'R1	TACGGTGCGACATATATTTCCCATTCGTATACCC
AvrPto1$_{Psy}$ 3'F2	AATATATGTCGCACCGTAACTGGTAATGA
AvrPto1$_{Psy}$ 3'R2	AGAAAGCTGGGTCTACAGCGACATGCTGGAACT
PTO-EcoRI-F	GGGAATCATGGGAAGCAAGTATTCTAA
PTO-SalI-R	AGCTTGTCGACAATAACAGACTCTTGGAGAC
PTO T204R-F	GTGAAAGGAAGACTCGGCTAC
PTO T204R-R	GTAGCCGAGTCTTCCTTTCAC
PTO T204N-F	GTGAAAGGAAATCTCGGCTAC
PTO T204N-R	GTAGCCGAGATTTCCTTTCAC
FEN-EcoRI-F	GGGAATTCATGGGAAGCAAGTATTCC
FEN-SalI-R	AGCTTGTCGACGTTCAGGATCATCTTGAATGG
FEN N202T-F	ACAGTAGTGAGAGGAACTATAGGCTACATTGCC
FEN N202T-R	GGCAATGTAGCCTATAGTTCCTCTCACTACTGT
AvrPto1$_{Psy}$-EcoRI-F	CCGAATTCGATGGGAAATATATGTGTC
AvrPto1$_{Psy}$-SalI-R	CTTGTCGACCCAGTTACGGTGCGGGCT
AvrPto1$_{Psy}$T91A-F	CAGCATAGGTTCATGGCGGGAGCGTCAGGA
AvrPto1$_{Psy}$T91A-R	TCCTGACGCTCCCGCCATGAACCTATGCTG
AvrPto1$_{Psy}$H125A/H130A-F	GAAGCTCTGCAGGCCGCAATGGGTATCGCCGCTGACATCCCA
AvrPto1$_{Psy}$H125A/H130A-R	TGGGATGTCAGCGGCGATACCCATTGCGGCCTGCAGAGCTTC
SlRIPK-SalI-F	CGGGTCGACATGGCTACTTGCGGAATTGACT
SlRIPK-Not1-R	GGGCGGCCGCTATAAGAATTTAGTTTGTTTG
SlRIN4-1-BamHI-F	GTGGATCCGATGGCTCGTCCAAATGTCCC
SlRIN4-1-XhoI-R	CCGGCTCGAGTTACCAGGGACAACAACACC
SlRIN4-2-BamHI-F	GTGGATCCGATGGCTCGTGCAAATGTG
SlRIN4-2-XhoI-R	CCGCTCGAGTCACCACAAAGGAAAGCA
SlRIN4-3-BamHI-F	GTGGATCCGATGGCAAAACACTCACAA
SlRIN4-3-XhoI-R	CCGCTCGAGTCAATCCGACCCCATGGAAACAT
AvrPto1$_{Psy}$-DONR-F	GGGGACAAGTTTGTACAAAAAAGCAGGCTTCATGGGAAATATATGT GTCGGC
AvrPto1$_{Psy}$-DONR-R	GGGACCACTTTGTACAAGAAAGCTGGGTCCCAGTTACGGTGCGGG CTAGG
PTO-DONR-F	AAAAAGCAGGCTTCATGGGAAGCAAGTATTCTAA
PTO-DONR-R	AGAAAGCTGGGTCAATAACAGACTCTTGGAGAC
FEN-DONR-F	AAAAAGCAGGCTTCATGGGAAGCAAGTATTCC
FEN-DONR-R	AGAAAGCTGGGTCTTCAGGATCATCTTGAATGG
SlRIPK-DONR-F	AAAAAGCAGGCTTCATGGCTACTTGCGGAATTGACT
SlRIPK-DONR-R	AGAAAGCTGGGTCATAAGAATTTAGTTTGTTTG
SlRIN4-1-DONR-F	AAAAAGCAGGCTATGGCTCGTCCAAATGTCCC
SlRIN4-1-DONR-R	AGAAAGCTGGGTCCCAGGGACAACAACACCA
SlRIN4-2-DONR-F	AAAAAGCAGGCTATGGCTCGTGCAAATGTG
SlRIN4-2-DONR-R	AGAAAGCTGGGTCCCACAAAGGAAAGCAGCA
SlRIN4-3-DONR-F	AAAAAGCAGGCTATGGCAAAACACTCACAA
SlRIN4-3-DONR-R	AGAAAGCTGGGTCACTCCGACCCCATGGGAA

Mutated codons are underlined.
