# Supplementary material for: Pseudomonas syringae effector HopZ3 suppresses the bacterial AvrPto1–tomato PTO immune complex via acetylation
Source: PLoS Pathog. 2021 Nov 1;17(11):e1010017. doi: 10.1371/journal.ppat.1010017 (PMC8584673; doi:10.1371/journal.ppat.1010017)
Supplement: S7 Fig — Modifications in SlRIN4s were determined in vitro, modifications in AtRIN4 are from [9] (in vitro and in planta). Residues acetylated by HopZ3 are bold and highlighted in yellow; phosphorylation sites are underlined; known phosphorylation sites important for signaling (S141, T166) in AtRIN4 [6] are highlighted blue; residues phosphorylated by RIPK in AtRIN4 (T21, S160, T166) [3] are circled in red. * (asterisk)—fully conserved residues,: (colon)—conservation between groups of strongly similar properties,. (period)—conservation between groups of weakly similar properties. (PDF) [file ppat.1010017.s007.pdf]

```
4_1     MAR-PNVPKFGNWENDDNTPYTVYFEKARQTRGT-GKMMNPNDPE---------EN----- 45
4_2     MAR-ANVPKFGNWGNEDNTPYTVVFENARKNRG--GKMINPNDPQ---------EN----- 44
4_3     MAKHSQVPKFGEWESDEDVQYTTYFENAAKGKK--GSKMNPNDPQYLEAKVKGENGTDTV 58
AT      MAR-SNVPKFGNWEAEENVPYTAYFDKARKTRAPGSKIMNPNDPE---------YN----- 46
        **: .:*****:*  :::. **. *::* : :   .. :*****:          *
4_1     ------------------------PDMFRNLAPPPEVAPQSKPKRQ--------TEEPPI 73
4_2     ------------------------PDMF------PNVAPSSRPKTP--------PTEEPM 66
4_3     RQKPERIASRDDVELRKSTGSPMHPDTMG-----HKVPTYPSPQRHGAKYGGNKSESETM 113
AT      ----------------------------------SDSQSQAPPHPP--------SSRTKP 64
                                           .  . . *:          .
4_1     GRGGPARQTRDHRLSKEDGEFRQYANSPARKESVGRKGANEPSHQR----GRGSNSGRTG 129
4_2     G-METARQTNKRRVSKEDGDFR--ASSPARNEPT-----THQRHGG----GRGSNSGRPS 114
4_3     KSTEILTPRHERRPSREEGYLRKPTDSPLRNENMGRRTPMESPHHRYGGLSGGATPKRAS 173
AT      EQVDTVRRSREHMRSREESELKQFGDAGG--------SSNEAANKR---------QGRAS 107
                 ..:  *:*:. ::   .:          .  :            *..
4_1     RQSIGSEHSFDKSPLHPHYQAKVSNAGRGVASPAWEGKN----------NSYDSSHGTPG 179
4_2     RQSGGSDHSIAKSPLHPNSQAKIS--GRVAASPVWEGKNLYDSSHGTPGRSFESSHATPG 172
4_3     QQSVGPDRSIEHSPLHPHSHGRPGGKGGVVSSPSWERKASSE-------GSHGLAPSTPG 226
AT      QNNSYDN----KSPLHKNSYDGTG-------------------------------KSRPK 132
        ::.   :    :**** :     .                                 . *
4_1     RSKVK-----QDKSDRGAAVPRFGEWDENDPQSADNYTHIFNKFREEKQGNPSGTPS--- 231
4_2     RHQMK-----QESPDRGTVVPKFGGWDDNDPQDAENYTEVFNKVREQRHVDTGNMPAAGV 227
4_3     RSRLRPVAKGDDTPDDSPAVPKFGDWDENDPASAEGYTQIFNKVREEKQTGSAKVPSSST 286
AT      PTNLR----ADESPEKVTVVPKFGDWDENNPSSADGYTHIFNKVREERSSGANVSGSSRT 188
          .::     ::..:  ..**:** **:*:* .*:.**.:***.**::  ..    :
4_1     RTSNNT-QKHNSEEKQRKWCCCPW--- 254
4_2     RTSYST-QRQQRNEKQKSCCFPLW--- 250
4_3     DTSYSNSQKRYGNDSGKGCLCFPWGRS 313
AT      PTHQSS---RNPNNTS-SCCCFGFGGK 211
         *  ..   :  ::.        :
```

S7 Fig. Alignment of tomato RIN4s (4_1, 4_2, 4_3) and Arabidopsis RIN4 (AT). Modifications in SlRIN4s were determined *in vitro*, modifications in AtRIN4 are from [1] (*in vitro* and *in planta*). Residues acetylated by HopZ3 are bold and highlighted in yellow; phosphorylation sites are underlined; known phosphorylation sites important for signaling (S141, T166) in AtRIN4 [2] are highlighted blue; residues phosphorylated by RIPK in AtRIN4 (T21, S160, T166) [3] are circled in red. * (asterisk) - fully conserved residues, : (colon) - conservation between groups of strongly similar properties, . (period) - conservation between groups of weakly similar properties.

References

1. Lee J, Manning AJ, Wolfgeher D, Jelenska J, Cavanaugh KA, Xu H, et al. Acetylation of an NB-LRR plant immune-effector complex suppresses immunity. Cell Rep. 2015;13(8):1670-82. doi: 10.1016/j.celrep.2015.10.029. PubMed PMID: 26586425.
2. Chung EH, El-Kasmi F, He Y, Loehr A, Dangl JL. A plant phosphoswitch platform repeatedly targeted by type III effector proteins regulates the output of both tiers of plant immune receptors. Cell Host & Microbe. 2014;16(4):484-94. doi: 10.1016/j.chom.2014.09.004. PubMed PMID: 25299334.
3. Liu J, Elmore JM, Lin ZJ, Coaker G. A receptor-like cytoplasmic kinase phosphorylates the host target RIN4, leading to the activation of a plant innate immune receptor. Cell Host & Microbe. 2011;9(2):137-46. doi: 10.1016/j.chom.2011.01.010. PubMed PMID: 21320696.
